# Recyclable Shape-Memory Waterborne Polyurethane Films Based on Perylene Bisimide Modified Polycaprolactone Diol

**DOI:** 10.3390/polym13111755

**Published:** 2021-05-27

**Authors:** Kang Wei, Haitao Zhang, Jianbo Qu, Jianyong Wang, Yang Bai, Futao Sai

**Affiliations:** 1 School of Light Industry Science and Engineering, Shandong Academy of Sciences, Qilu University of Technology, Jinan 250353, China; wk180203@163.com (K.W.); qujianbo396@sohu.com (J.Q.); wjy@qlu.edu.cn (J.W.); 17864181422@163.com (F.S.); 2 State Key Laboratory of Biobased Material and Green Papermaking, Shandong Academy of Sciences, Qilu University of Technology, Jinan 250353, China; 3 Shaanxi Key Laboratory of Chemical Additives for Industry, College of Chemistry and Chemical Engineering, Shaanxi University of Science and Technology, Xi’an 710021, China

**Keywords:** perylene bisimide, waterborne polyurethane, polycaprolactone diol, recyclable, shape-memory

## Abstract

Currently, much attention is given to the functionality and recyclability of waterborne polyurethane (WPU). Herein, ε-caprolactone was used as a chain extender for grafting onto perylene bisimide (PBI) and 1,4-butanediol (BDO) via ring-opening reactions to obtain PBI-PCL and BDO- PCL. Then, two kinds of WPU, namely PBI-WPU (PWPU) and BDO-WPU (BWPU), were fabricated using PBI-PCL/polytetrahydrofuran ether glycol (PTMG) and BDO-PCL/PTMG, respectively, as mixed soft segments. The properties and appearance of PWPU and BWPU emulsions were analyzed in terms of particle size, zeta potential and TEM images, and the results showed that PWPU emulsions had uniform particle size distribution and decent storage stability. AFM and DMA results revealed that PWPU films possessed a more significant degree of microphase separation and a higher glass transition temperature (T_g_) than BWPU films. The PWPU films displayed good shape-memory and mechanical properties, with tensile strength up to 58.25 MPa and elongation at break up to 1241.36%. TGA analysis indicated that PWPU films had better thermal stability than BWPU films. More importantly, the PWPU films could be dissolved in a mixed solvent of acetone/ethanol (*v*/*v* = 2:1) at room temperature. The dissolved PWPU could be dispersed in deionized water to prepare waterborne polyurethane again. After the recycling process was repeated three times, the recycled PWPU emulsion still exhibited good storage stability. The recycled PWPU films maintained their original thermal and mechanical properties. Comparing the properties of BWPU and PWPU showed that the soft segment structure had important influence on waterborne polyurethane performance. Therefore, PWPU may have great potential applications in making recycling and shape-memory coating or paint.

## 1. Introduction

Polyurethane can be classified into waterborne polyurethane (WPU) and solvent-based polyurethane (SPU), based on the dispersion media. WPU and SPU have many comparable properties, such as high tensile strength, high abrasion resistance, high flexibility, good adhesion and good low-temperature resistance [1,2]. The difference between WPU and SPU is that the production and use of WPU does not release a large amount of volatile organic compounds (VOC), which is an inevitable problem in the production and use of SPU [3,4]. Therefore, as an environmentally friendly material in the industry, waterborne polyurethane dispersions have supplanted SPU in the fields of synthetic leather, leather finishing, coating, textile laminating and adhesives [5,6]. WPU, including WPU coatings, is the most important material today. Hence, their functional modification and recycling have been of great interest. Polyurethane is recycled in two primary ways: mechanical/physical recycling and chemical recycling. The mechanical/physical recycling method is generally done to process polyurethane waste into other usable products through compression molding, adhesive pressure molding, extrusion molding, etc. [7]. Chemical recycling takes polyurethane back to its various chemical constituents [8]. However, the recycling process mentioned above has insufficient versatility for recycling polyurethane composite materials. For instance, synthetic leather is mainly composed of polyurethane coating, and the base fabric is composed of chemical fiber. It is challenging to recycle base fabric through thermal processing. The separation of decomposition products of polyurethane and base fabric is even more difficult [9]. Therefore, it is vital to develop a new recycling process or a new waterborne polyurethane to improve the recyclability of polyurethane composite materials.

Solvent-based recycling has been widely used in recycled plastic products [10]. Such polyurethane treatment is challenging due to its complex crosslinking structure. Wang et al., prepared a series of waterborne polyurethanes (WPU-DA-x) using Diels–Alder (DA) diol as a chain extender. WPU-DA-x films show excellent self-healing capability due to the couple/decouple process of the DA reactions. Although WPU-DA-x films can be recycled via hot-pressing, although only by raising the dissolution temperature, these could be dissolved into DMF and recycled via solution casting [11]. Another solvent-based recycling method has been developed utilizing a polar and aprotic solvent for PU dissolution. PU was added to dimethylsulfoxide (DMSO), n-methyl pyrrolidone (NMP) or dimethylformamide (DMF). Then, a nonsolvent was added to the PU solution to form a suspension of PU. In the next step, the solvent was removed to form a PU dispersion for further treatment [12]. However, the recycling processes using DMSO, NMP and DMF solvents are all costly and not environmentally friendly. In the industrial production of waterborne polyurethane, a low-boiling solvent (like acetone) is commonly used to adjust the viscosity of the prepolymer. After the prepolymer is dispersed in water, the added low-boiling solvent can easily be removed by vacuum distillation [12]. Therefore, developing a WPU film which can be dissolved in low-boiling solvent, and the recycled solution can then be dispersed by water again, is very useful for green recycling of waterborne polyurethane.

Shape-memory waterborne polyurethane has attracted significant attention due to its potential application in various fields, such as sensors, smart textiles and medical implants. Poly(ε-caprolactone) (PCL) diol is crystallizable, biodegradable and widely used for the soft segments of PU [13]. The crystallization and phase behavior of PCL strongly affect the shape-memory property of PU [14]. In a previous study, Czifrák et al., synthesized block polyurethane using 50 kDa PCL, 1,6-hexamethylene diisocyanate (HDI) and poly(ω-pentalactone) (PPDL) as raw materials to prove that the segmented structure may fulfill the requirement for the construction of shape-memory polymers (SMPs) [15]. Yang and his colleagues synthesized a new shape-memory polyurethane network (A-SMPUs) by using PCL as the soft segment, 4,4-azodibenzoic acid (Azoa) and hexamethylene diisocyanate (HDI) as the hard segments, and chemically crosslinking with glycerol (Gl). A-SMPUs have a microphase separation structure composed of a semicrystalline PCL soft phase and azo amorphous hard phase, showing good triple-shape-memory performance [16]. Naddeo et al., reported that the complementary characteristics of PLA and PCL have suggested taking advantage of both through the copolymerization of the respective monomers. Promising shape-memory properties are also observed in di-block copolymers characterized by the co-crystallization of CL and L-LA segments [17]. The melting/crystallization of PCL is employed for utilizing the reversible switching function of its shape-memory behavior.

In short, the preparation of waterborne polyurethane with environmentally friendly recyclability and shape-memory versatility is of great significance. However, there are relatively few studies on such waterborne polyurethane. Therefore, to prepare multifunctional waterborne polyurethane, this paper presents work that attempts to graft polycaprolactone on both ends of rigid PBI and use it as a soft segment to prepare waterborne polyurethane materials. Perylene bisimide (PBI) and its derivatives are organic dyes with excellent optical properties and thermal stability. PBI-modified polyurethane has attracted much attention in terms of enhanced shape-memory, mechanical properties and thermal stability [18,19]. For instance, Li and his co-workers prepared a series of amphiphilic PBI-Pus using PBI oligomers and HDI. The thermal stability of the PBI-Pus was higher than that of neat PU [20]. Additionally, Lu et al., reported that, compared to the samples without PBI, the PSMP (prepared by PCL as the soft segment and PBI as chain extender) with 5.5 wt.% PBI showed the highest tensile properties and good shape-memory properties [21]. However, these reported PBI-modified PUs are all solvent-based polyurethanes. The effect of PBI on WPU has not yet been reported.

## 2. Materials and Methods

### 2.1. Materials

1,7-dibromo-3,4,9,10-perylene tetracarboxylic anhydride (PBI) was obtained from Zhengzhou Alpha Chemical Co. Ltd. (Zhengzhou, China). Polytetramethylene ether glycol (PTMG, *M*_n_ = 2000) was obtained from Beijing Branch, Du Pont China Holding Co. Ltd. (Beijing, China) and was degassed and dried under high vacuum (0.8 mmHg) at 120 °C overnight before use. Isophorone diisocyanate (IPDI), ethanolamine, 6-caprolactone, 1,4-butanediol (BDO), stannous octoate, 2,2-bis (hydroxymethyl) propionic acid (DMPA) and triethylamine (TEA) were all purchased from Shanghai Macleans Biochemical Technology Co. Ltd. (Shanghai, China). Organic bismuth catalyst and 2-[(2-aminoethyl) amino]-ethanesulfonic acid monosodium salt (A95) was procured from Sinopharm Chemical Reagent Co. Ltd. (Shanghai, China). The solvents ethanol, acetone and N-methyl-2-pyrrolidone (NMP) were of analytical grade, provided by Shanghai Aladdin Biochemical Technology Co. Ltd. (Shanghai, China). The solvent toluene was of analytical grade, provided by Sinopharm Chemical Reagent Co. Ltd. (Shanghai, China). Toluene was freshly distilled before use.

### 2.2. Preparation of PWPU_-X_ and BWPU_-X_ Emulsions

**Scheme 1:**
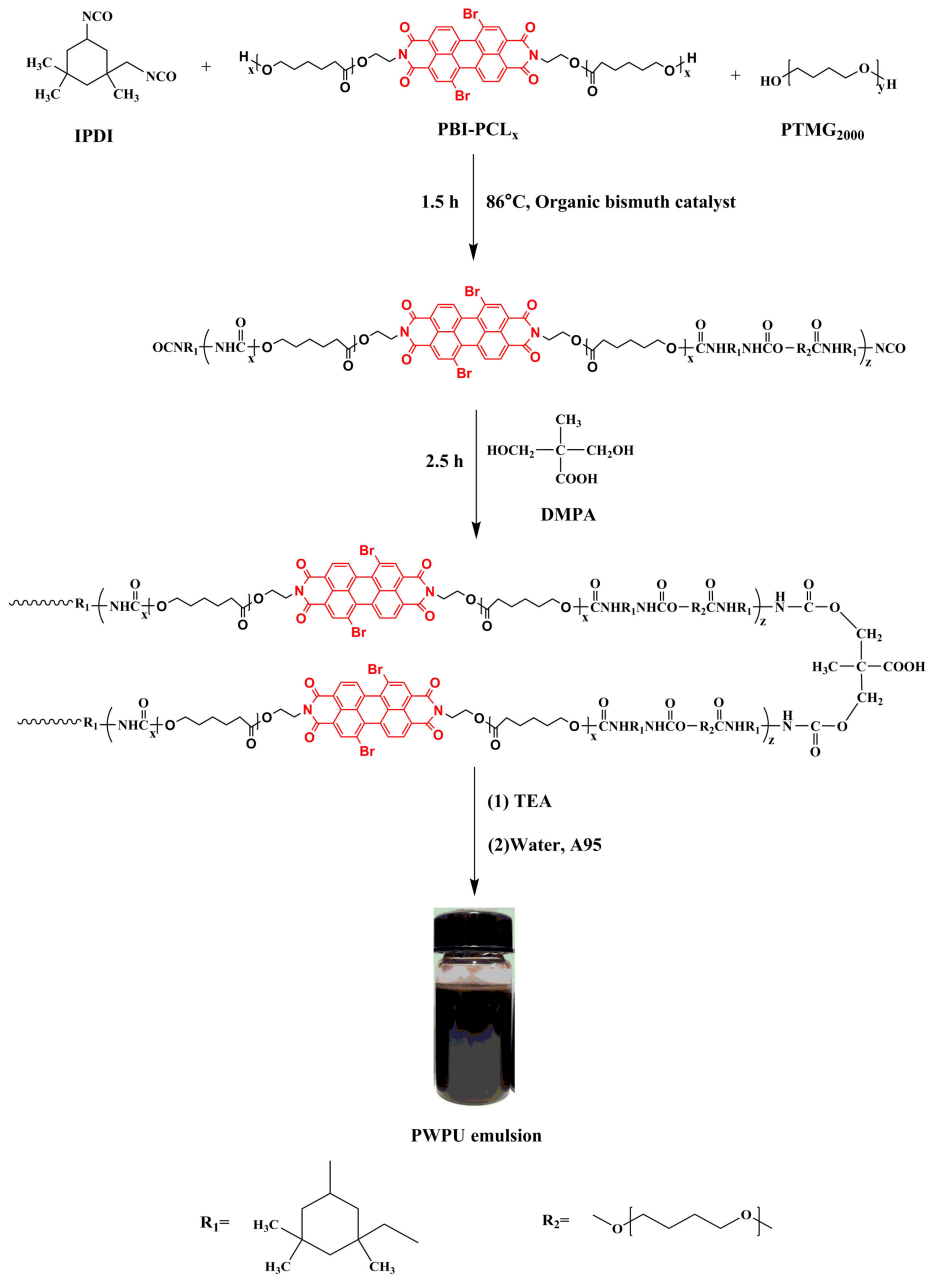
Synthetic route of PWPU_-X_ emulsion.

PBI-PCL_-X_, with molecular weights of 5000 and 10,000 Da, were prepared by ring-opening polymerization of caprolactone, using PBI as the initiator. As shown in Scheme 1, a series of waterborne polyurethanes, namely PWPU_-X_ with fixed PTMG and different amounts of PBI-PCL as soft segments, were prepared using the prepolymer method. PBI-PCL, PTMG and IPDI reacted for 1.5 h under the catalysis of organic bismuth at 86 °C. Then, DMPA was added to continue the reaction for another 2.5 h. After the reaction completed, the temperature was reduced to 45 °C, and triethylamine was added for about ten minutes to neutralize the medium. Then, the reaction solution was sheared and dispersed in water, and A95 was added into the system as a post-chain extender. Finally, a PWPU_-X_ emulsion was obtained. As control, BDO-modified polycaprolactone diol BDO-PCL with molecular weight of 5000 and 10,000 Da and waterborne polyurethane BWPU_-X_ with fixed PTMG and different amounts of BDO-PCL as soft segments, were prepared through the same method. The detailed synthetic process is shown in the ESI, Section 1.

### 2.3. Fabrication of Films

The WPU films were prepared by pouring the dispersion into a polytetrafluoroethylene (PTFE) plate, and dried under ambient conditions for 48 h. The films were then peeled from the PTFE plate and dried at 60 °C for 24 h in a vacuum-drying oven to completely remove the moisture.

### 2.4. Characterization

^1^H-NMR spectra were measured with a 400 MHz Bruker instrument. Chemical shifts were reported in parts per million (ppm), and CDCl_3_ was used as a solvent for all the samples. Fourier transform infrared (FTIR) spectra were obtained using a Shimadzu IR Affinity-1s spectrometer(Shimadzu Co., Kyoto, Japan). Each sample was analyzed in the range of resolution from 400 to 4000 cm^−1^ with a spectral resolution of 4 cm^−1^, and 32 scans were collected.

Emulsion performance tests were performed on a Zetasizer Nano ZS90(Malvern Instruments Ltd., Worcestershire, UK) under the following conditions: test concentration was configured to 0.5 mg/mL, test temperature was 25 °C, and the final results were the average of three measurements.

TEM was performed on a JEM-2100F electron microscope(JEOL, Tokyo, Japan) operating at 200 kV. The test conditions were as follows: A drop of polyurethane emulsion with solid content of 0.05% was taken on a copper grid and precipitated for 30 min, excess liquid was blotted off on filter paper; then, the samples were stained with 2% phosphotungstic acid for 10 min. The excess liquid was blotted off by filter paper and frozen with liquid nitrogen, and the freeze-dryer was tested after 3 h.

Thermal stability analysis was determined with a Mettler Toledo Instruments thermogravimetric analyzer (Mettler-Toledo, Columbus, OH, USA) under a nitrogen atmosphere. Each sample (~10 mg) was heated from 25 °C to 650 °C at a rate of 10 °C/min.

The glass transition temperature (T_g_) of the samples was determined under a TA Instruments Q800 Dynamic Thermodynamic Analyzer (TA Instruments, Hüllhorst, Germany). The samples (about 5 mg) were heated from −100 °C to 100 °C at a rate of 5 °C/min.

Stress-strain curves were measured using a universal testing machine (High-speed machine VWN-20, Hongxing Machinery Co., Guangdong, China) at room temperature with a tensile speed of 100 mm/min.

The AFM phase diagram of the polyurethane film was tested in tapping mode using a multimode 8 AFM device (multimode 8, Bruker, Santa Barbara, USA). The scanning range was 20 × 20 μm^2^.

## 3. Results and Discussion

### 3.1. Synthesis of PWPU_-X_ and BWPU_-X_ Emulsions

**Figure 1:**
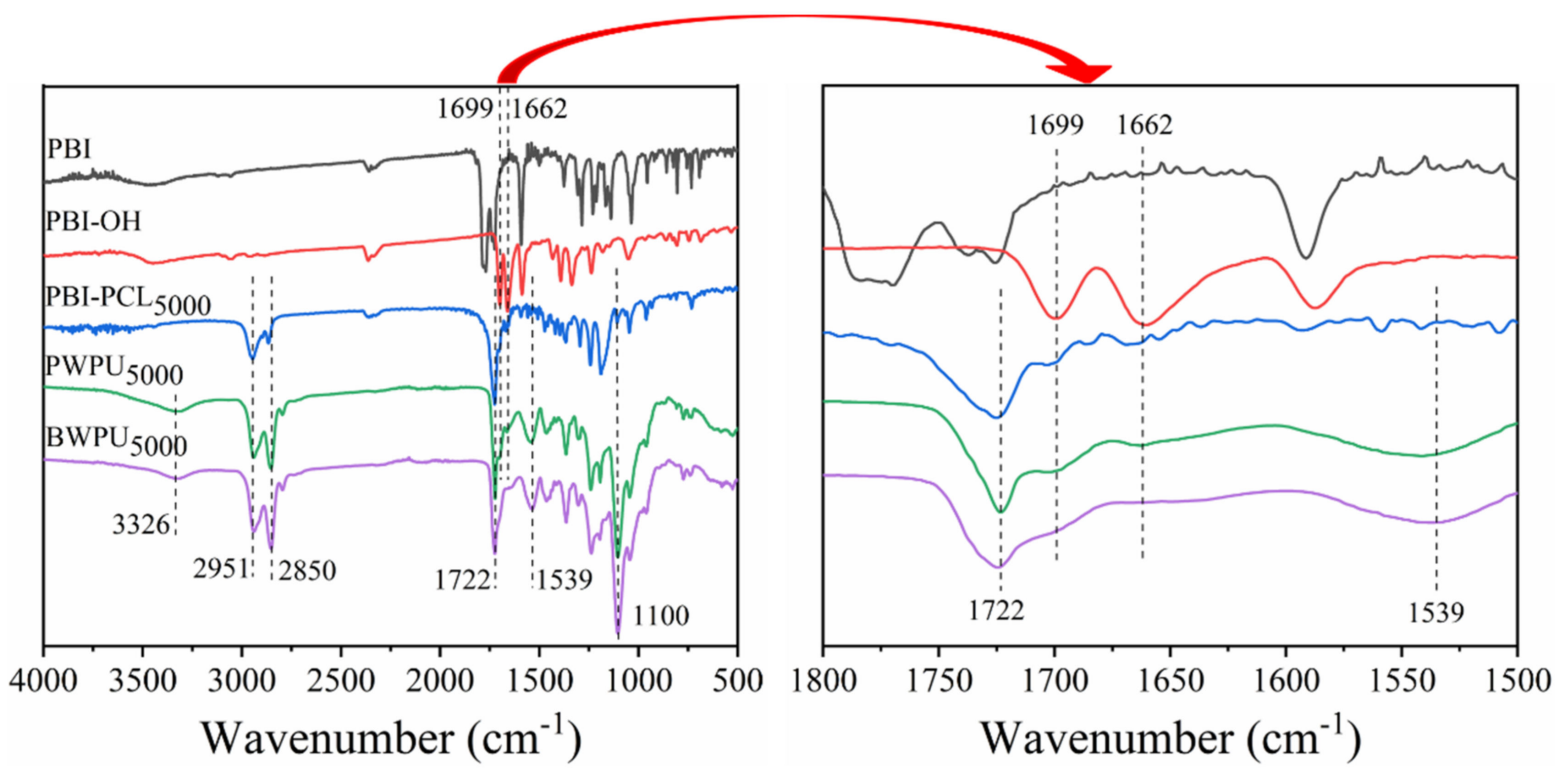
FTIR spectra of PBI, PBI-OH, PBI-PCL_5000_, PWPU_5000_ and BWPU_5000_.

Various studies have reported that tailoring the construction and constants of soft segments is a simple and valuable technique to design properties of polyurethanes [22,23,24,25]. Therefore, a series of novel soft segments, PBI-PCL_5000_, PBI-PCL_10,000_, BDO-PCL_5000_ and BDO-PCL_10,000_, were prepared by ring-opening polymerization (ESI, Section 1). Those soft segments’ polymer structure and molecular weight were confirmed through proton nuclear magnetic resonance structures (^1^H-NMR) (Figures S1 and S2). Then, waterborne polyurethane, namely PWPU_5000_, PWPU_10,000_, BWPU_5000_ and BWPU_10,000_, were prepared using PBI-PCL/PTMG and BDO-PCL/PTMG as mixed soft segments, respectively (Scheme 1, Table 1, Schemes S1–S4). Figure 1 shows the FTIR spectra of PBI, PBI-OH, PBI-PCL_5000_, PWPU_5000_ and BWPU_5000_. The spectra of PWPU_5000_ and BWPU_5000_ showed strong peaks at 3326 cm^−1^, assigned to the –N–H stretching vibration in the urethane group. The peaks at 1539 cm^−1^ and 1722 cm^−1^ denote the –N–H bond stretching and stretching vibrations of –C=O bond, respectively. The band at 1100 cm^−1^ denotes the characteristic absorption of –C–O–C stretching vibrations in PTMG. Moreover, a shoulder peak of the –C–H band in PTMG and PCL was observed at 2951 cm^−1^ and 2850 cm^−1^. The strong band at 1699 cm^−1^ and 1662 cm^−1^ belongs to the –C–N vibrations in PBI-OH, and it is recognizable in PBI-PCL_5000_ and PWPU_5000_. Figure S3 displays the FTIR spectra of BDO, BDO-PCL_10,000_, BWPU_10,000_ and PWPU_10,000_ (Supporting Information). These results confirmed the successful preparation of PWPU_-X_ and BWPU_-X_.

### 3.2. Properties of PWPU_-X_ and BWPU_-X_ Emulsions

**Figure 2:**
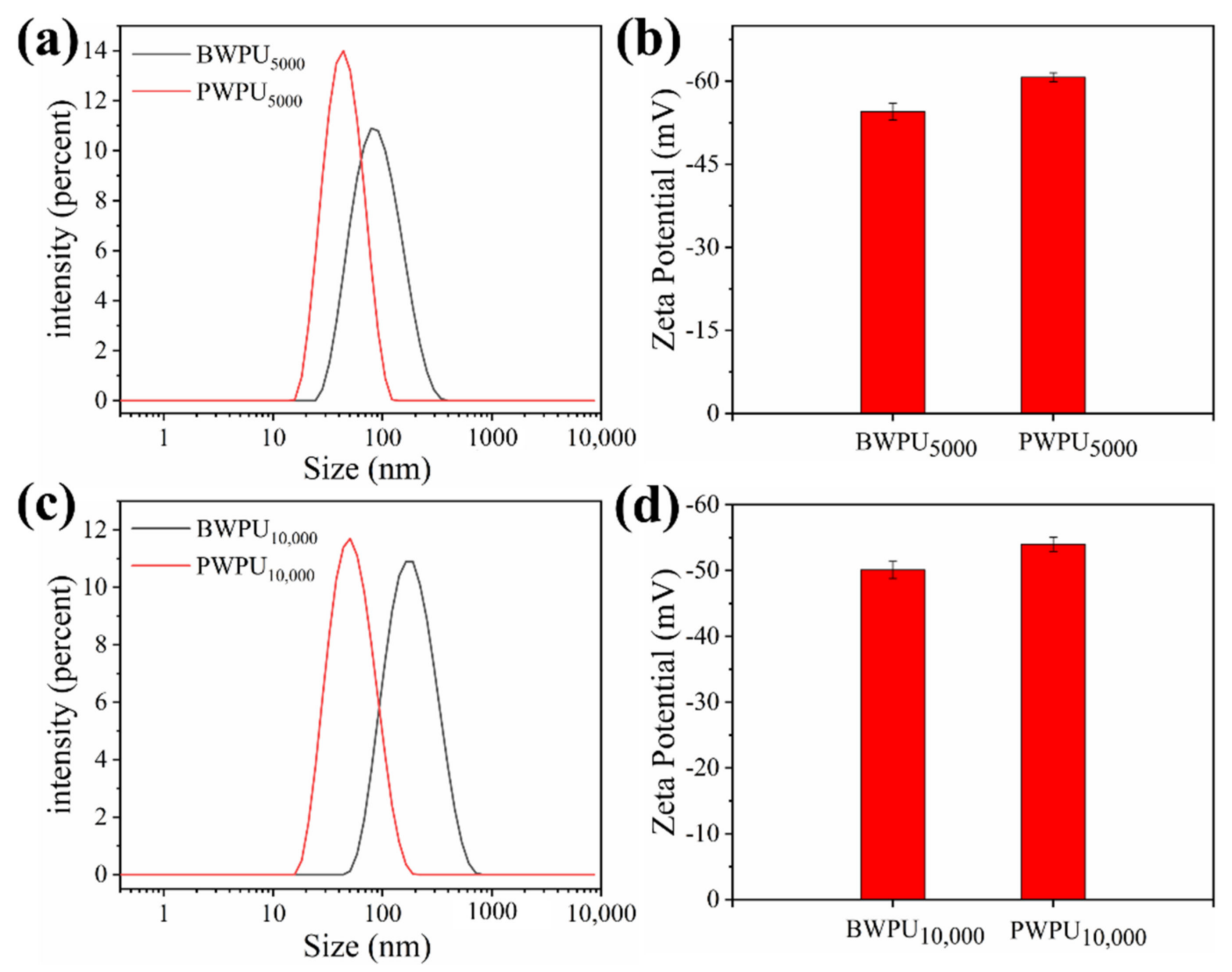
(**a**) Particle size and distribution of BWPU_5000_ and PWPU_5000_; (**b**) zeta potential of BWPU_5000_ and PWPU_5000_ emulsions; (**c**) Particle size and distribution of BWPU_10,000_ and PWPU_10,000_; (**d**) zeta potential of BWPU_10,000_ and PWPU_10,000_ emulsions.

DLS results were used to evaluate the storage stability of a WPU dispersion [26]. The Z-average diameter (*D*z) and the size distribution of BWPU_5000_, BWPU_10,000_, PWPU_5000_ and PWPU_10,000_ are shown in Table 2. The *D*_Z_ of PWPU_5000_ and PWPU_10,000_ were 40.26 nm and 49.05 nm, respectively (Figure 2a,c). The sizes of PWPU_10,000_ particles were greater than those of PWPU_5000_, and similar results could be found in BWPU_10,000_ and BWPU_5000_ (Figure 2a,c). These results indicate that the *D*z of WPU increased with the increase in the soft segment length, which follows the result reported by Parashar. P et al. [27]. Moreover, PWPU_5000_ and PWPU_10,000_ showed smaller particle sizes than BWPU_5000_ and BWPU_10,000_, respectively. the reason may be as follows: The introduction of PBI reduced the flexibility of the soft segments, reducing the mutual entanglement between the soft segments in the prepolymer, making the prepolymer easier to disperse and assemble in water under shearing action. The number of polyurethane segments in each polyurethane emulsion particle was reduced. The particle size was reduced. The DLS results indicate that PBI-embedded soft segments PBI-PCL was expected to facilitate the dispersion of the waterborne polyurethane prepolymer.

Furthermore, zeta potential is another important indicator for evaluating the stability of the emulsions [28]. A higher value of zeta potential confers stability due to the strong repulsion between the emulsion particles. The zeta potentials of BWPU_5000_, PWPU_5000_, BWPU_10,000_ and PWPU_10,000_ emulsions were −54.5 mV, −60.7 mV, −50.1 mV and −54.0 mV (Figure 2b,d and Figure S4), respectively. With a similar soft segment length and formula, the absolute value of the zeta potential of PWPU was higher than that of BWPU, demonstrating the enhanced stability of PWPU emulsions. Combined with the DLS results (Figure 2b,d), the increase in zeta potential was probably because the incorporation of PBI reduced the particle size of the emulsion. Hence, the density of carboxyl groups per unit volume of emulsion particle increased. In other words, the charge per unit volume increased, thereby causing the increase in the absolute value of zeta potential [29].

**Figure 3:**
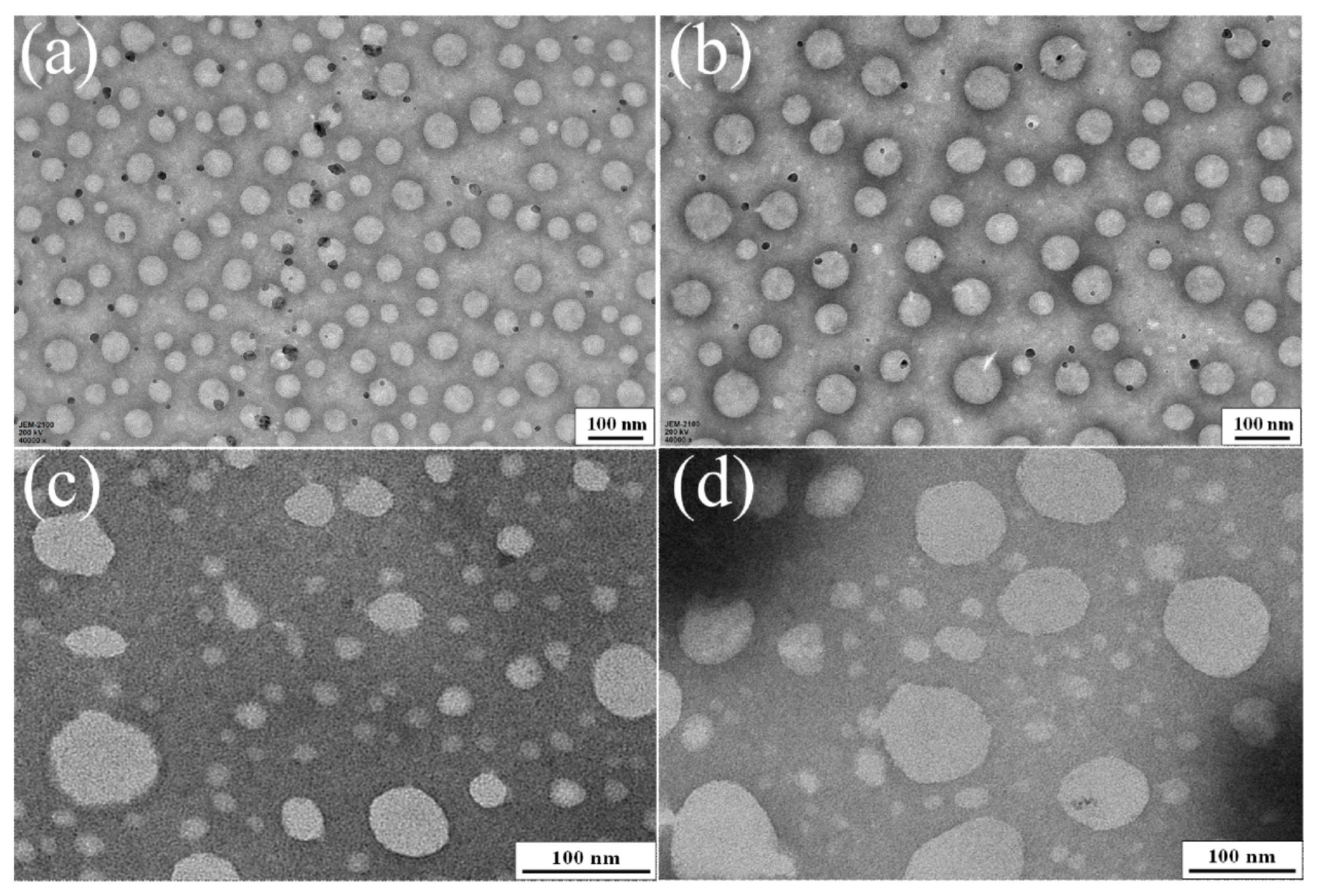
TEM images of PWPU and BWPU. (**a**) PWPU_5000_; (**b**) PWPU_10,000_; (**c**) BWPU_5000_; (**d**) BWPU_10,000_; (concentration 0.5 mg/mL).

The morphology of BWPU_5000_, BWPU_10,000_, PWPU_5000_ and PWPU_10,000_ emulsions were investigated by TEM. As depicted in Figure 3a,b, the particle size of PWPU_5000_ and PWPU_10,000_ emulsions from TEM observation were 41 nm and 45 nm, which is consistent with the results measured by DLS. As depicted in Figure 3c,d, the particle size of BWPU_5000_ and BWPU_10,000_ emulsions from TEM observation were 72 nm and 115 nm, consistent with the results measured by DLS. These results further illustrate that embedding PBI in the soft segments is expected to provide better stability for PWPU emulsions.

### 3.3. Mechanical and Thermal Performance

**Figure 4:**
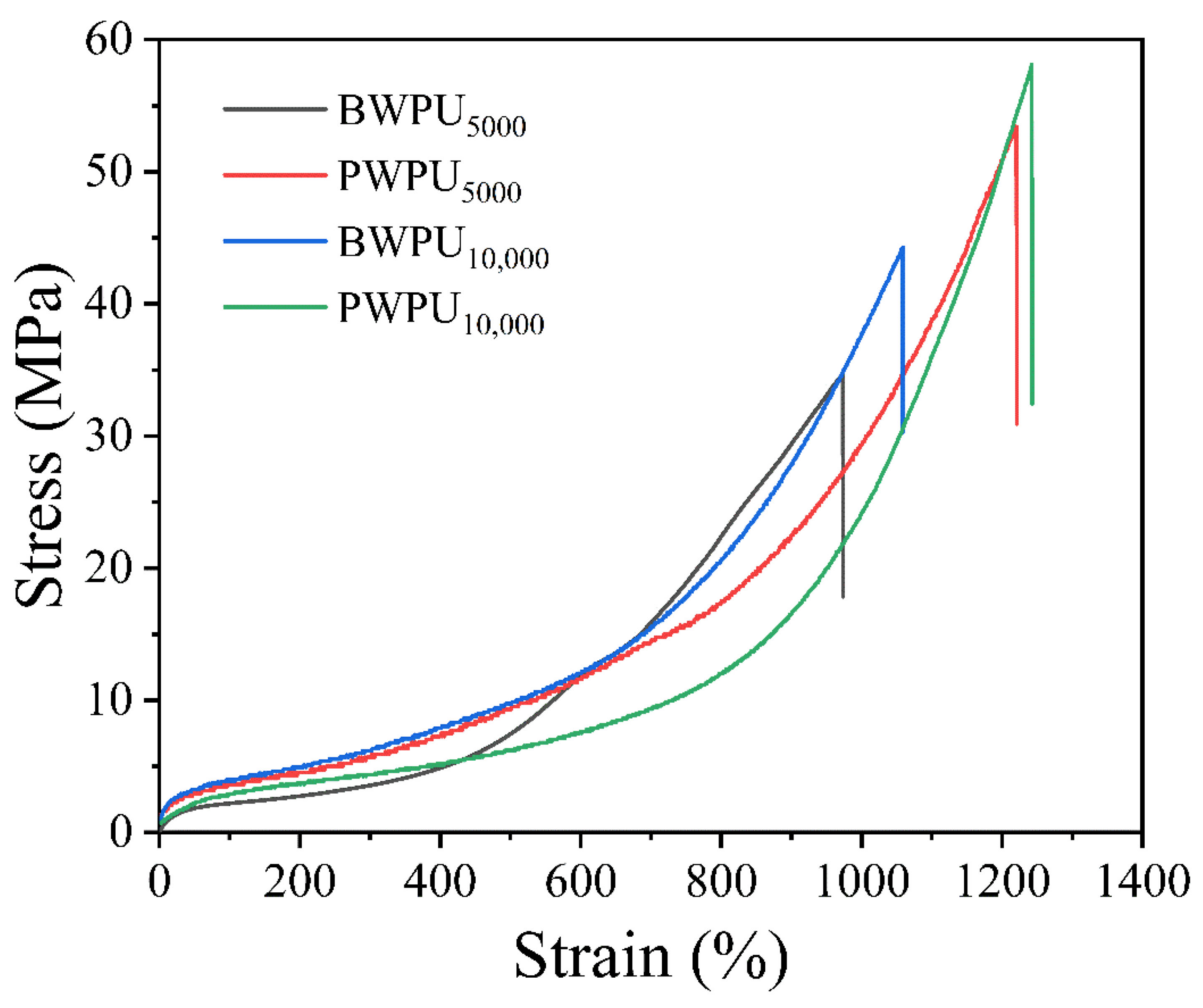
Stress-strain curves of BWPU_5000_, BWPU_10,000_, PWPU_5000_ and PWPU_10,000_ films.

**Figure 5:**
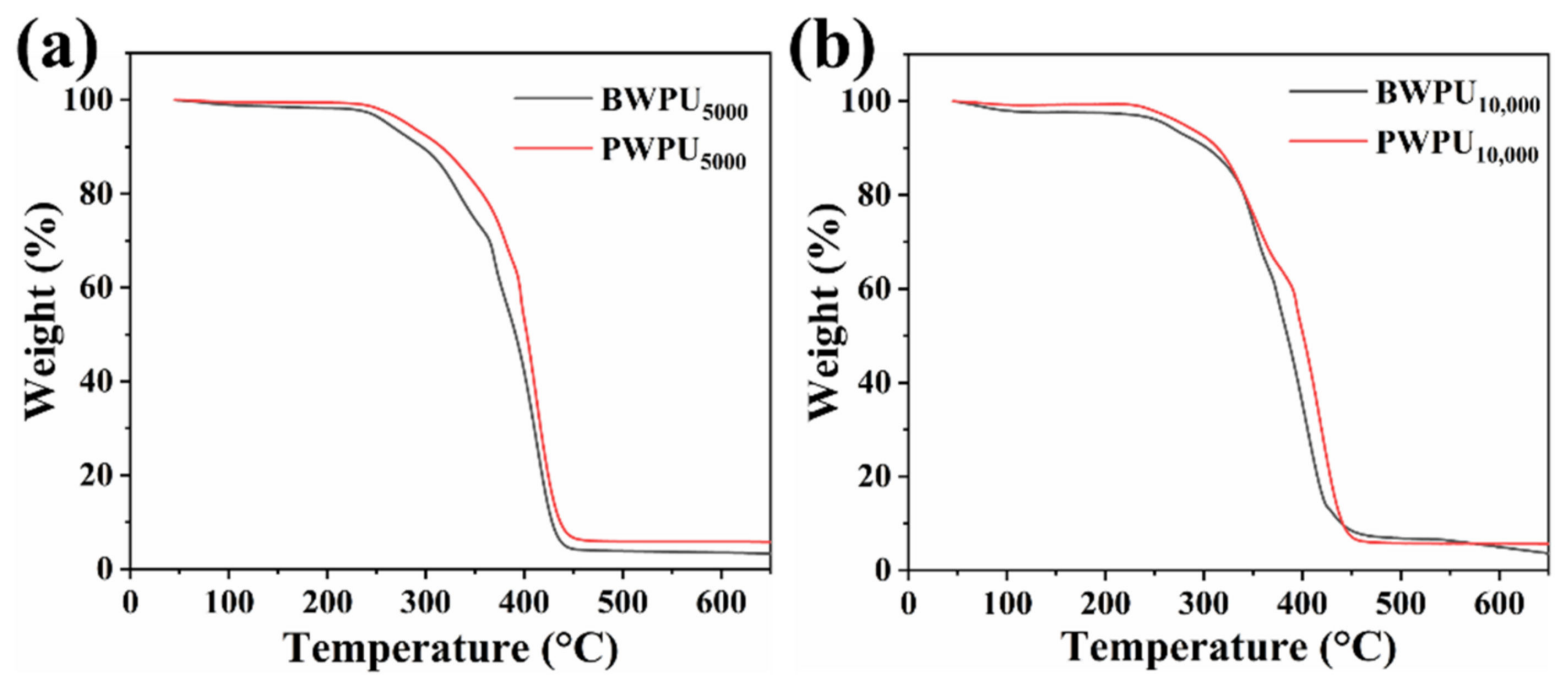
(**a**) TGA curves of BWPU_5000_ film and PWPU_5000_ film; (**b**) TGA curves of BWPU_10,000_ film and PWPU_10,000_ film.

The stress-strain properties of BWPU_5000_, BWPU_10,000_, PWPU_5000_ and PWPU_10,000_ films were tested using a tensile testing machine. As shown in Figure 4, PWPU_5000_ and PWPU_10,000_, whose fracture stresses were 53.58 MPa and 58.25 MPa, respectively, displayed higher strength. In contrast, the fracture stresses of BWPU_5000_ and BWPU_10,000_ were 35.02 MPa and 44.3 MPa, respectively. Additionally, the breaking elongation of PWPU films was larger than that of the BWPU films. The high strength and breaking elongation of PWPU could be ascribed to the rigid structure of PBI, which increased the cohesive energy of the polyurethane and the strengthened intermolecular forces. TGA was employed to further assess the effect of the addition of PBI-PCL and BDO-PCL on the thermal stability of PWPU and BWPU films. The thermograms of BWPU_5000_, BWPU_10,000_, PWPU_5000_ and PWPU_10,000_ films are shown in Figure 5. Table 3 summarizes the results of the selected parameters obtained from the TGA thermograms. PWPU films and BWPU films showed similar TGA curves, indicative of similar thermal degradation behaviors. The degradation curves of waterborne polyurethanes exhibit two stages. The temperature corresponding to 5% mass loss (T_5%_) is considered to be the onset of the WPU decomposition process [30]. The temperature of 50% mass loss (T_50%_, the second degradation temperature) corresponds to the temperature range of the decomposition of soft segments [30]. The T_5%_ of PWPU_5000_ and PWPU_10,000_ were 281.0 °C and 280.1 °C, higher than those of BWPU_5000_ and BWPU_10,000_, respectively (Table 3). The value of T_50%_ increased from 390.3 °C to 402.7 °C as the soft segment BDO-PCL_5000_ was replaced by PBI-PCL_5000_. The value of T_50%_ increased from 384.3 °C to 401.2 °C when the soft segment BDO-PCL_10,000_ was replaced by PBI-PCL_10,000_ (Table 3). These results suggest that substitution of BDO with PBI in the soft segment enhanced the thermal stability of the WPU films, because the amide group and aromatic ring in PBI can form hydrogen bonds with carbamate and improve the attraction between the soft segments and hard segment of WPU [31].

### 3.4. AFM and DMA Analysis

#### 3.4.1. AFM Analysis

**Figure 6:**
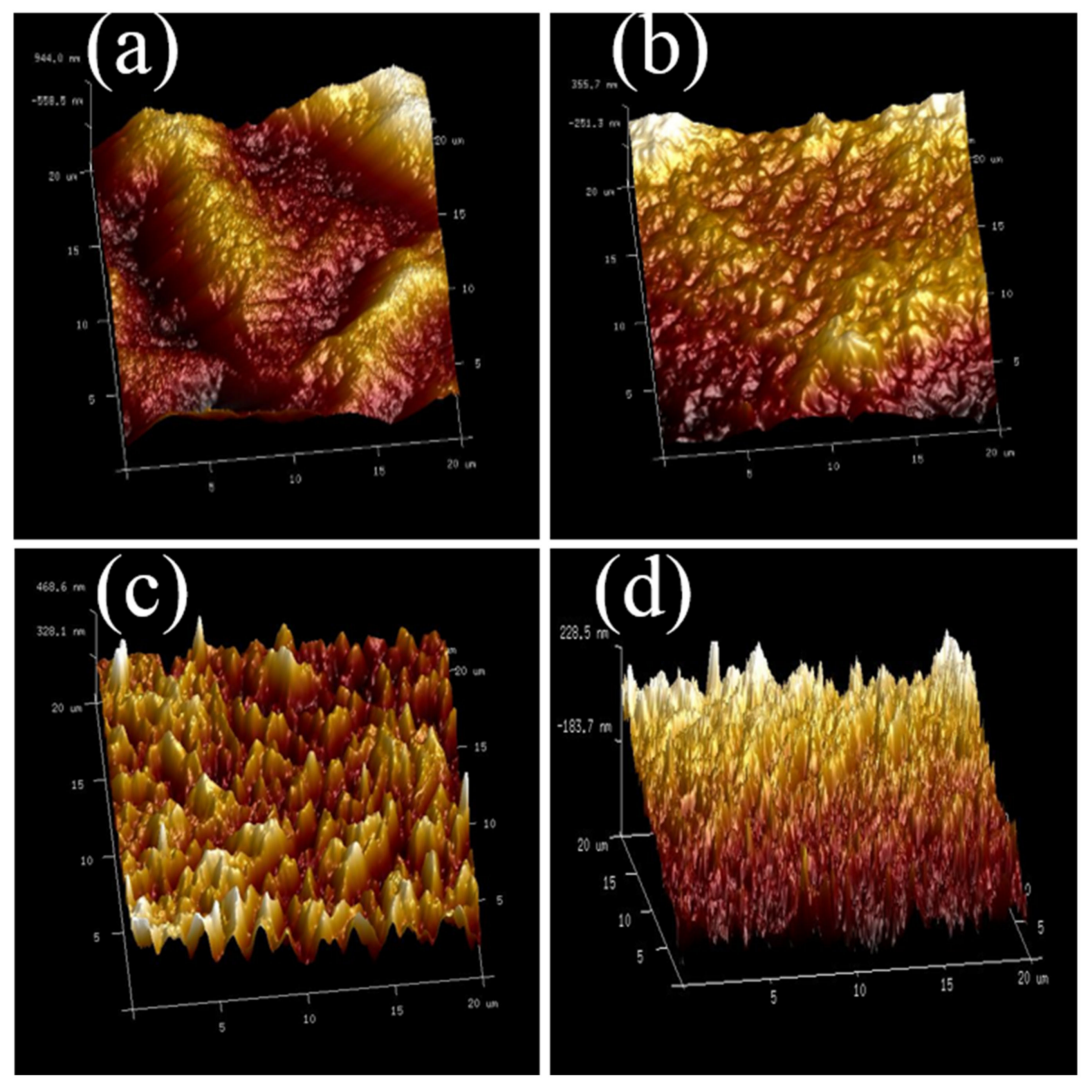
3D morphology images of BWPU_5000_ (**a**), BWPU_10,000_ (**b**), PWPU_5000_ (**c**) and PWPU_10,000_ (**d**).

**Figure 7:**
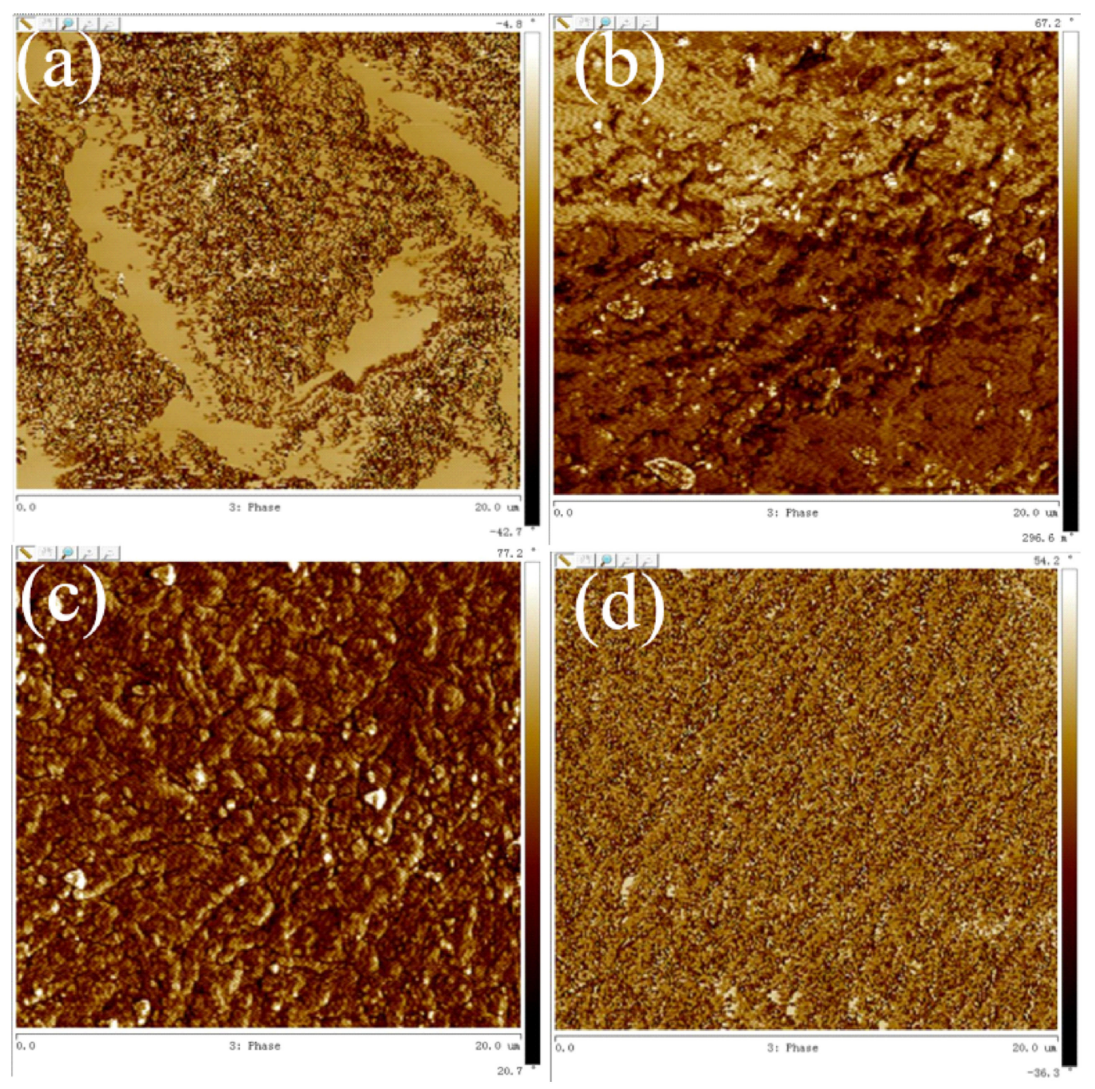
AFM phase images of BWPU_5000_ (**a**), BWPU_10,000_ (**b**), PWPU_5000_ (**c**) and PWPU_10,000_ (**d**).

The degree of microphase separation considerably affects many physical properties of polyurethane [32]. The microphase-separated structures of the PWPU and BWPU films were investigated using AFM and DMA. Initially, we used AFM surface technology to investigate the phase difference between BWPU and PWPU. As presented in Figure 6 and Figure 7, the bright part corresponds to the polyurethane hard section, and the concave dark part corresponds to the polyurethane soft section [33]. PWPU and BWPU films exhibited an evidently uneven surface, suggesting a phase separation structure. As one can see, the size and dispersion of microphase-separated structure seemed to be dissimilar depending on the soft segments. For instance, in the sample of BWPU and PWPU, when the soft segment BDO-PCL_5000_ was substituted by PBI-PCL_5000_, the size of domains decreased, clearly dispersed phases were observed, and more regular microphase separation appeared (Figure 6a,c, and Figure 7a,c). Similar phase change was observed in the samples of BWPU_10,000_ and PWPU_10,000_ (Figure 6b,d and Figure 7b,d). As shown in Figure 6, we also found that the surface roughness of PWPU was higher than that of BWPU. The foremost reason may also be that microphase separation in PWPU was better than in BWPU. The results of phase images (Figure 7) further confirmed that introducing rigid PBI in the soft segment played a significant role in the formation of microphase separation. Following the augmentation of surface roughness, the contact area with the solvent was also increased, leading to a faster dissolution time, so that the recycling process of PWPU was easier. The recycling process for PWPU and BWPU will be compared in the following part.

#### 3.4.2. DMA Analysis

**Figure 8:**
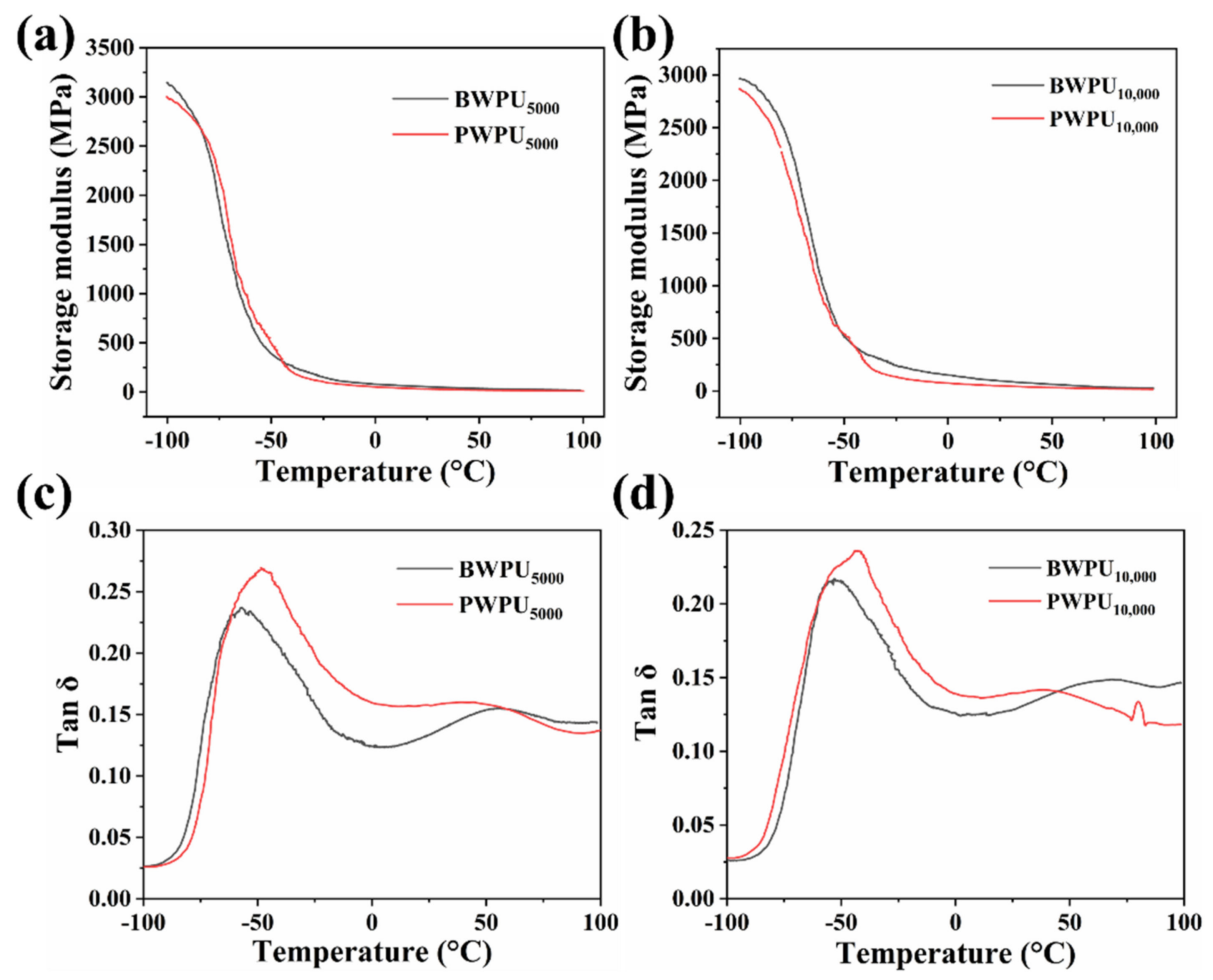
(**a**) Storage modulus versus temperature in BWPU_5000_ and PWPU_5000_; (**b**) Storage modulus versus temperature in BWPU_10,000_ and PWPU_10,000_; (**c**) Tan δ versus temperature in BWPU_5000_ and PWPU_5000_; (**d**) Tan δ versus temperature in BWPU_10,000_ and PWPU_10,000_.

To further investigate the effects of the soft segment structure on the microphase separation of waterborne polyurethanes, DMA was employed to further investigate the phase changes of PWPU and BWPU films. Storage modulus (E′) curves of BWPU_5000_, BWPU_10,000_, PWPU_5000_ and PWPU_10,000_ are shown in Figure 8a,b. The variation in storage modulus (E´) reflects the effect of temperature on the activity of the soft and hard segments of polyurethane. The storage modulus is related to the degree of ordering in the polymer structure. As the temperature increases, the regular accumulation of molecules becomes problematic, and the storage modulus decreases. For PWPU and BWPU films, the storage modulus decreases sharply at temperatures between −100 °C and −50 °C, indicating that the polyurethane undergoes a phase change in this temperature range [34]. The reason for these results is that the PWPU and BWPU soft segments, which are largely composed of highly reactive PTMG and PCL (both -C-C and -C-O-C), have strong mobility at low temperatures, making molecular chain activity more sensitive at increasing temperatures.

The mechanical loss tangent (Tan δ) can significantly reflect the glass transition temperature (T_g_) and microphase separation structure of polyurethane [35]. Figure 8c,d displays the Tan δ curves of PWPU and BWPU films. The Tan δ curves of all the WPU films show two peaks, indicating the existence of microphase separation structures in the internal structures of PWPU and BWPU films [34]. The peak at a lower temperature transition (range from −61 °C to −51 °C) may correspond to the release of a constrained amorphous phase. The peak at a high-temperature transition (range from 40 °C to 50 °C) was assigned to the liberation of polymer chains in the crystalline regions. The gaps between the two peaks of PWPU films were smaller than those of BWPU films, indicating that the compatibility between amorphous phase and crystalline regions was improved by the addition of PBI in PCL segments. Moreover, the PWPU films exhibited a much larger Tan δ peak compared to that of the BWPU films. The temperature associated with the peak magnitude of Tan δ was defined as the glass transition temperature (T_g_). The first T_g_ of BWPU_5000_ film, PWPU_5000_ film, BWPU_10,000_ film and PWPU_10,000_ film were −56.8 °C, −47.7 °C, −52.5 °C and −41.6 °C, respectively. The first T_g_ increased with the increase in soft segment, and the first T_g_ of PWPU was found to be higher than that of BWPU. Compared to BWPU films, PWPU films exhibited a broader Tan δ transition, suggesting a greater degree of nonuniformity of cross-links. Moreover, Dawn M. Crawford discovered that nonuniformity during film formation may introduce voids in the polymer matrix, allowing a greater number of solvent molecules to penetrate [36]. Therefore, the DMA result of BWPU and PWPU provides some insight into the fact that the solubility of WPU could be altered by tailoring the soft segment structure.

### 3.5. Recyclable Performance Analysis

#### 3.5.1. Green Recycling Process of PWPU_-X_ Films

**Figure 9:**
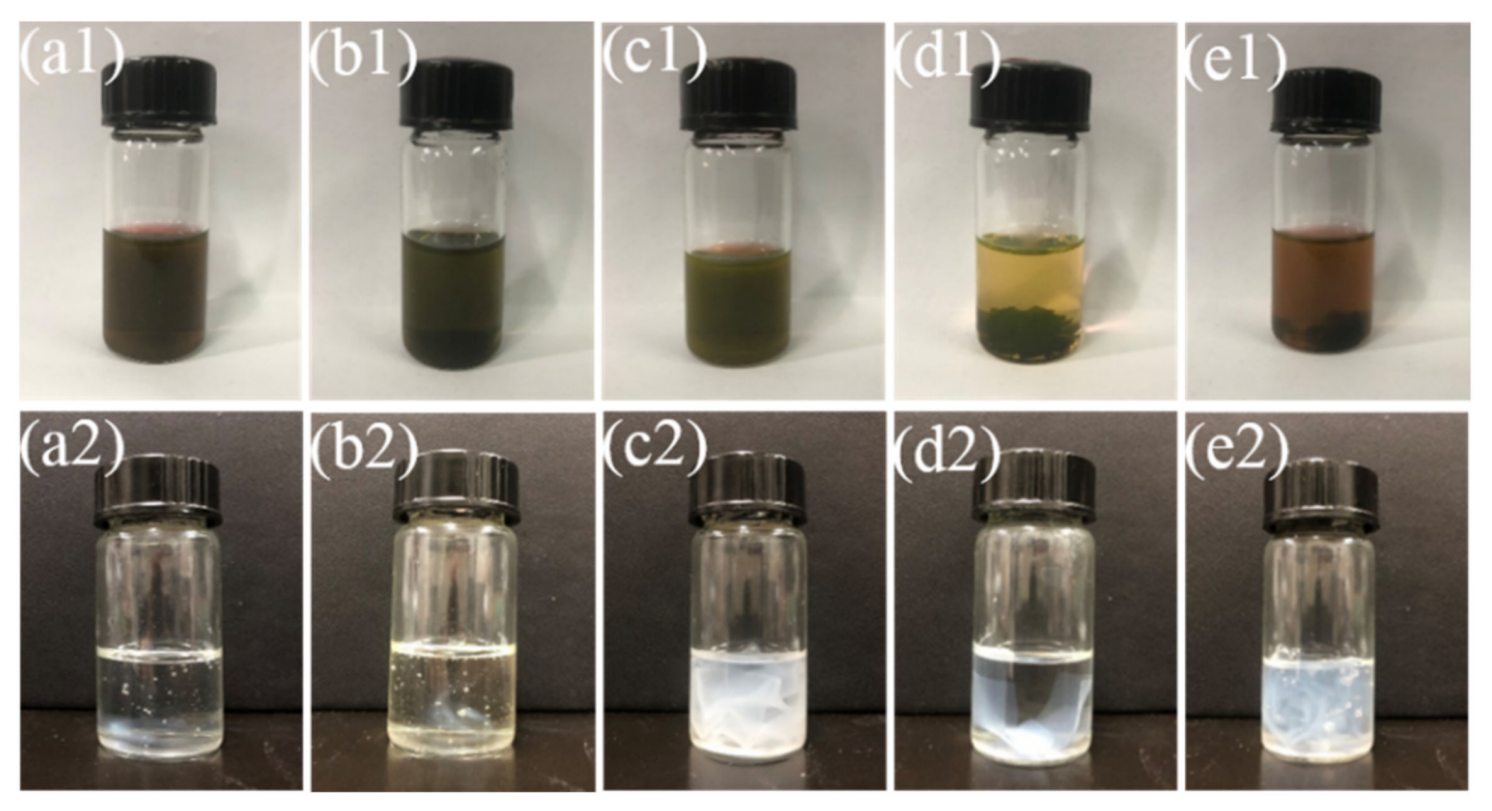
Photos of PWPU_5000_ with a mass of 0.5 g soaking in different solvents (4 mL) at 25 °C for 3 h. (**a1**) DMF, (**b1**) NMP, (**c1**) acetone/ethanol (*v*/*v* = 2:1), (**d1**) acetone, (**e1**) ethanol; Photos of BWPU_5000_ with a mass of 0.5 g soaking in different solvents (4 mL) at 25 °C for 3 h. (**a2**) DMF, (**b2**) NMP, (**c2**) acetone/ethanol (*v*/*v* = 2:1), (**d2**) acetone, (**e2**) ethanol.

The dissolution behavior of PWPU_5000_ and BWPU_5000_ in different solvents at 25 °C is shown in Figure 9. The PWPU_5000_ film exhibited the best dissolution ability, and it dissolved completely within 3 h when the solvent was acetone/ethanol (*v*/*v* = 2:1) or DMF (Figure 9(a1,a2,c1,c2)). However, although BWPU_5000_ film could be dissolved in polar solvents, DMF and NMP, it only swelled in other solvents and was not completely dissolved even after three days. The same dissolution results emerged in the PWPU_10,000_ and BWPU_10,000_ films (Figures S5 and S6). DMA and AFM results indicate that PWPU films exhibited a higher degree of nonuniformity of cross-links and a lot of voids for solvents to penetrate. This may be the reason behind PWPU films showing a preferable swelling and solubility behavior (Figures S7 and S8). Considering the eco-friendly feature of waterborne polyurethanes and the fact that acetone and ethanol can be removed by vacuum distillation, we used acetone/ethanol (*v*/*v* = 2:1) as the solvents for the recycling purpose.

**Figure 10:**
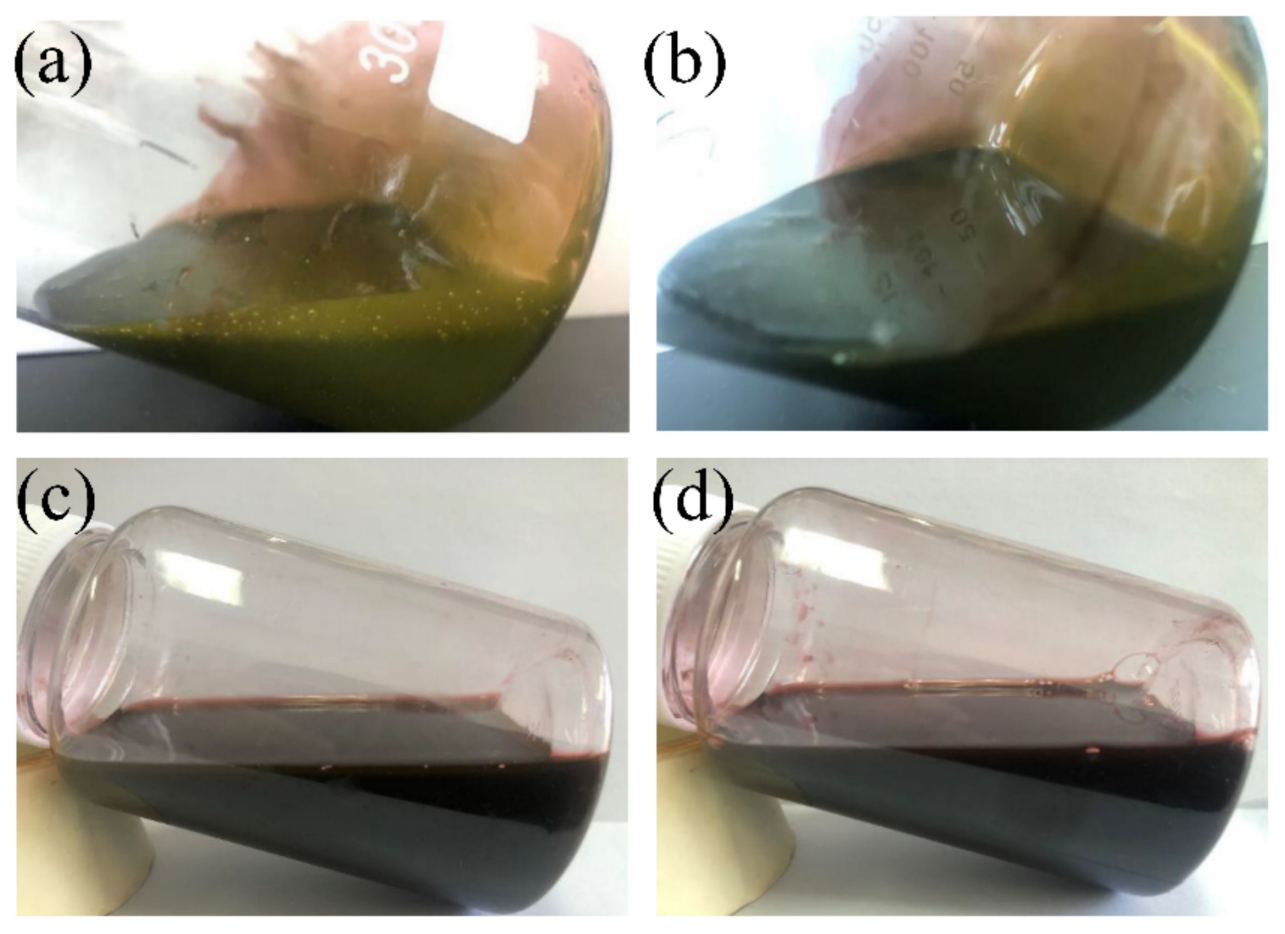
(**a**,**b**) Photos of PWPU_5000_ and PWPU_10,000_ with a mass of 10 g dissolved in 80 mL acetone/ethanol (*v*/*v* = 2:1) mixed solvent respectively at 25 °C for 3 h; (**c**,**d**) Photos of PWPU_5000_ and PWPU_10,000_ recycled emulsions.

The full recycling process of PWPU was as follows: Firstly, the chopped PWPU_5000_ and PWPU_10,000_ films (10 g) were dissolved in 80 mL of acetone/ethanol (*v*/*v* = 2:1) at room temperature. These were completely dissolved in 3 h, as shown in Figure 10a,b. Then, the dissolved mixture was directly dispersed in water using a high-speed shearing machine. After that, the acetone/ethanol solvent mixture was removed by vacuum distillation to obtain the recycled PWPU emulsion (Figure 10c,d). Finally, the recycled emulsion was poured into a PTFE plate to prepare a PWPU film.

**Scheme 2:**
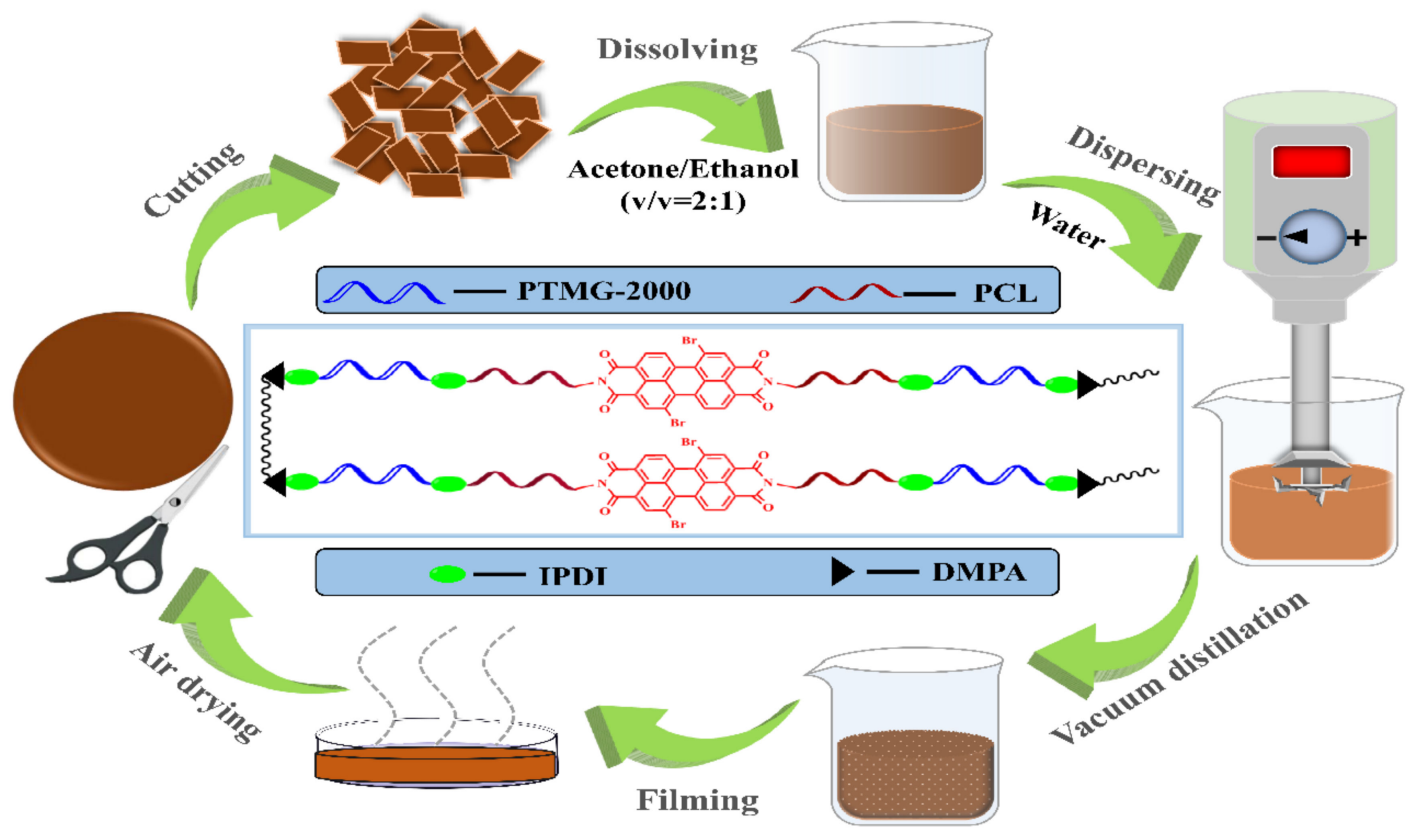
Schematic diagram of PWPU film recycling.

The green recycling process of PWPU film is shown in Scheme 2. The process primarily included six parts: (1) Film cutting; (2) dissolving the pieces of the film in the solvent mixture of acetone/ethanol (*v*/*v* = 2:1); (3) shearing and dispersing in water; (4) vacuum distillation to remove the organic solvents; (5) film formation; (6) drying to obtain the PWPU film. The whole process was carried out at room temperature and eventually, multiple green recycling of PWPU film was realized.

#### 3.5.2. Properties of Recycled PWPU_-X_ Emulsion

**Figure 11:**
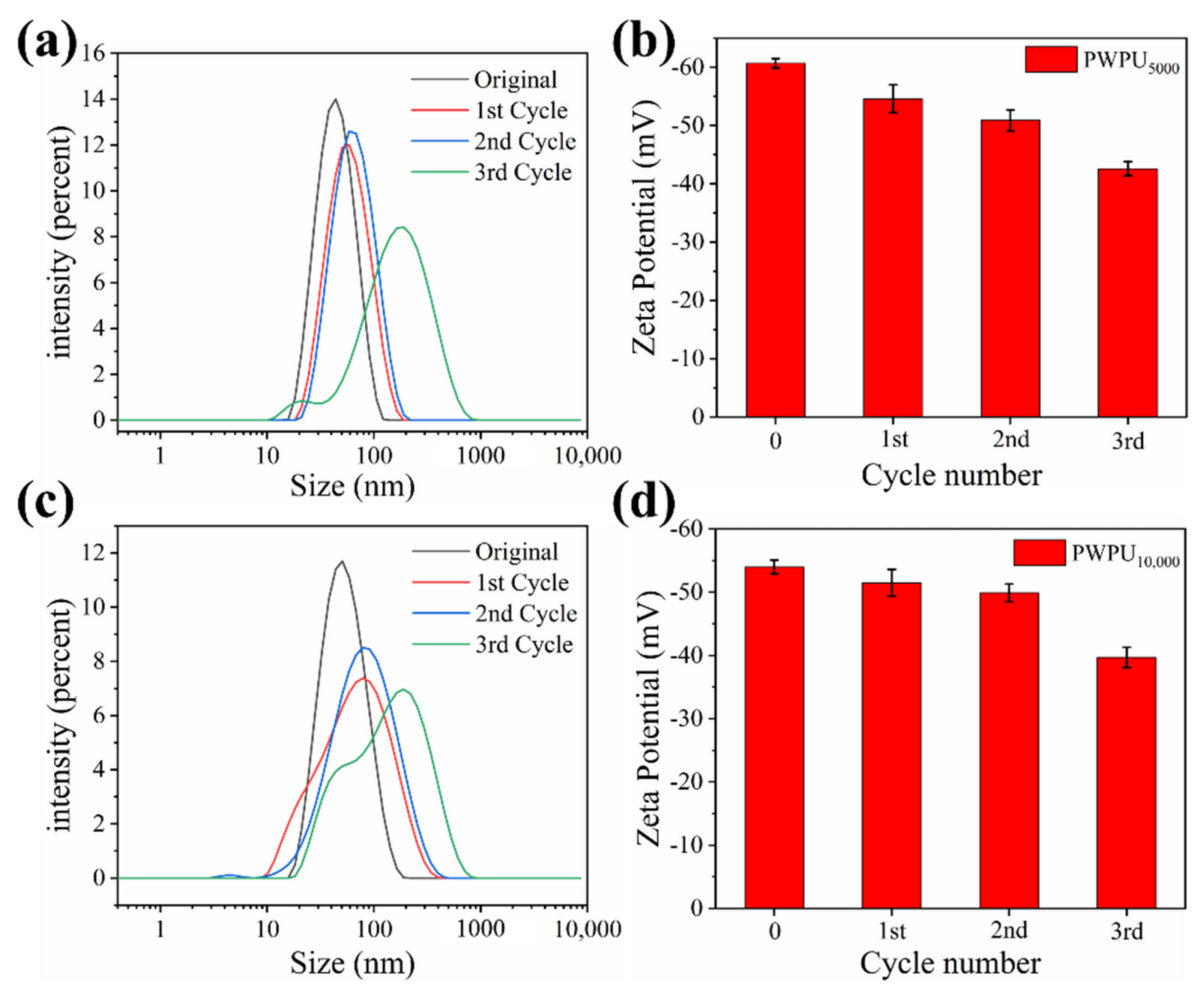
(**a**) Particle size and distribution of recycled PWPU_5000_ emulsions; (**b**) zeta potential of recycled PWPU_5000_ emulsions; (**c**) Particle size and distribution of recycled PWPU_10,000_ emulsions; (**d**) zeta potential of recycled PWPU_10,000_ emulsions.

The storage stability of recycled PWPU emulsions was evaluated by DLS. The Z-average diameter (*D*z) and the size distribution of recycled PWPU emulsions are shown in Figure 11. The *D*z of PWPU_5000_ emulsion increased from 40.26 to 97.95 nm. The size of PWPU_10,000_ emulsion increased from 49.05 to 122.02 nm after the recycling process was repeated three times (Figure 11a,c). The key reasons are as follows: The particle size of waterborne polyurethane is primarily related to the hydrophilicity of the chain segment. After triethylamine neutralizes dimethylolpropionic acid, the chain segment contains the carboxylate group. Ionization of carboxy group makes the prepolymer have certain hydrophilicity and accomplishes the purpose of self-emulsification in dispersing. Triethylamine is highly volatile, which could volatilize following the evaporation of water. The degree of ionization for carboxy group is subsequently reduced. Thus, as recycling cycles increase, the hydrophilicity of the system gradually decreases, leading to increased particle size. The size distribution of recycled emulsion gradually increases, and the absolute value of zeta potential in the system decreases. In the meantime, the absolute values of zeta potential of PWPU_5000_ and PWPU_10,000_ recycled emulsions decreased from 60.7 to 42.6 mV and from 54.0 to 39.7 mV, respectively (Figure 11b,d and Figure S9). Although the *D*z of recycled emulsions increase gradually, the absolute values of zeta potential of recycled emulsions were higher than 39 after being recycled three times. Moreover, zeta potential is the parameter which determines the stability of particles. Consequently, the PWPU emulsions still retain excellent stability after three recycling processes.

#### 3.5.3. Mechanical and Thermal Performance of Recycled PWPU_-X_ Film

**Figure 12:**
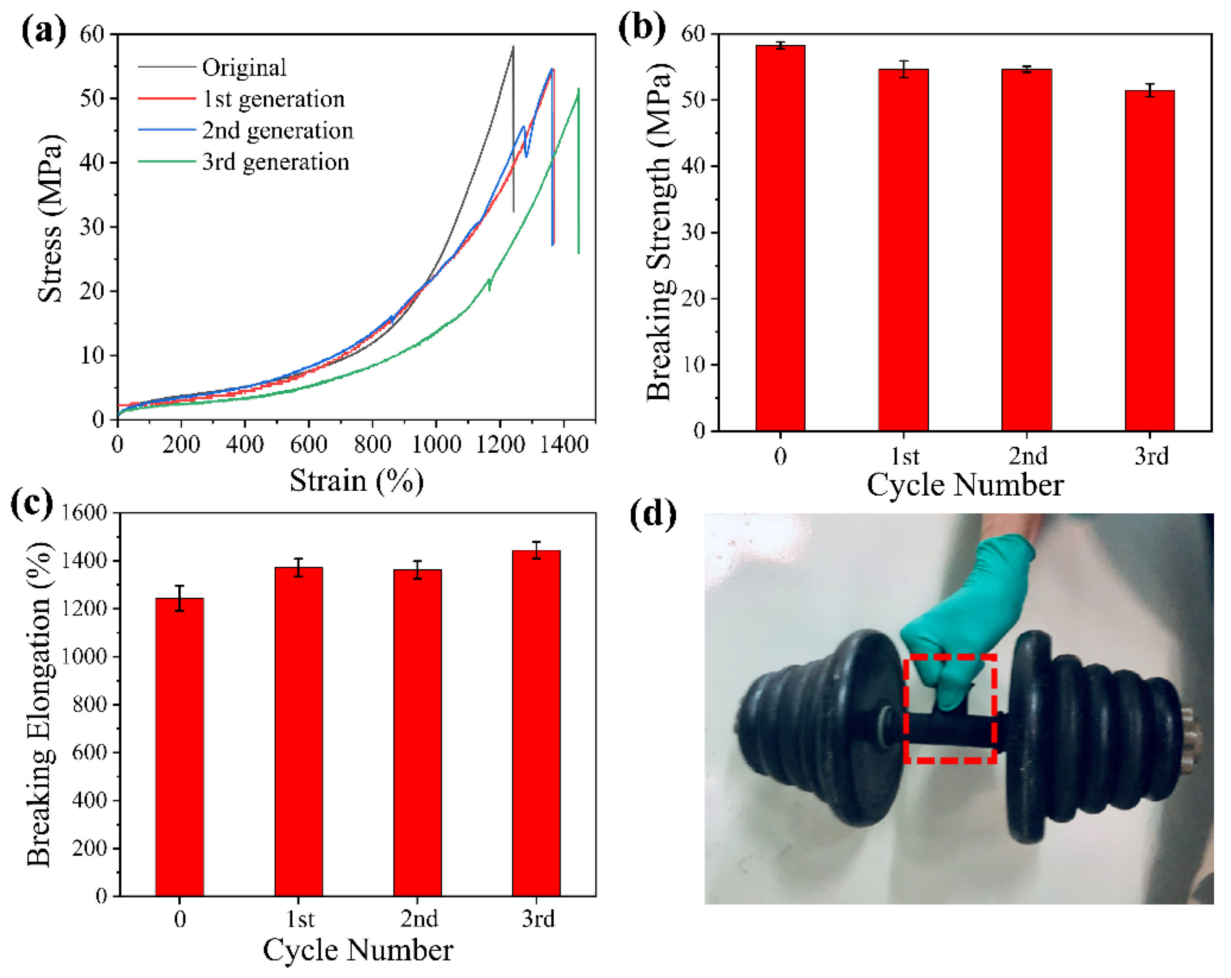
(**a**) Stress–strain curves of PWPU_10,000_ after three generations of recycling. (**b**) Breaking strength and (**c**) breaking elongation of the PWPU_10,000_ samples after three recycling processes. (**d**) Photo of lifting a 10 kg weight with a PWPU_10,000_ film with the thickness of 0.3 mm after the recycling process.

To further reveal the mechanical properties of reprocessed PWPU samples, tensile tests were performed. As shown in Figure 12, the recovered PWPU could keep the uniform stress-strain curve of the original sample (Figure 12a). More notably, as shown in Figure 12b,c, the tensile strength of PWPU_10,000_ decreased from 58.25 to 51.49 MPa as the breaking elongation increased from 1243.95% to 1443.86% after being recycled three times. These results indicated that the recovery rates of breaking strength and the breaking elongation of PWPU_10,000_ were larger than 89%. Similar findings were observed for PWPU_5000_ (Figure S10), indicating excellent recyclability of PWPU. Although other research groups recycled waterborne polyurethane by hot-pressing [37], solution casting [11] (dissolved by DMF or other high-boiling organic solvents) and other traditional polymer-processing methods [38], these methods typically have to be implemented accompanied by negative effects, such as operational difficulty, high-cost, environmental pollution and health hazards. Tensile strength and breaking elongation were significantly reduced after recycling multiple times. However, PWPU still exhibited remarkable mechanical properties as well as outstanding recyclability after reusing multiple times.

**Figure 13:**
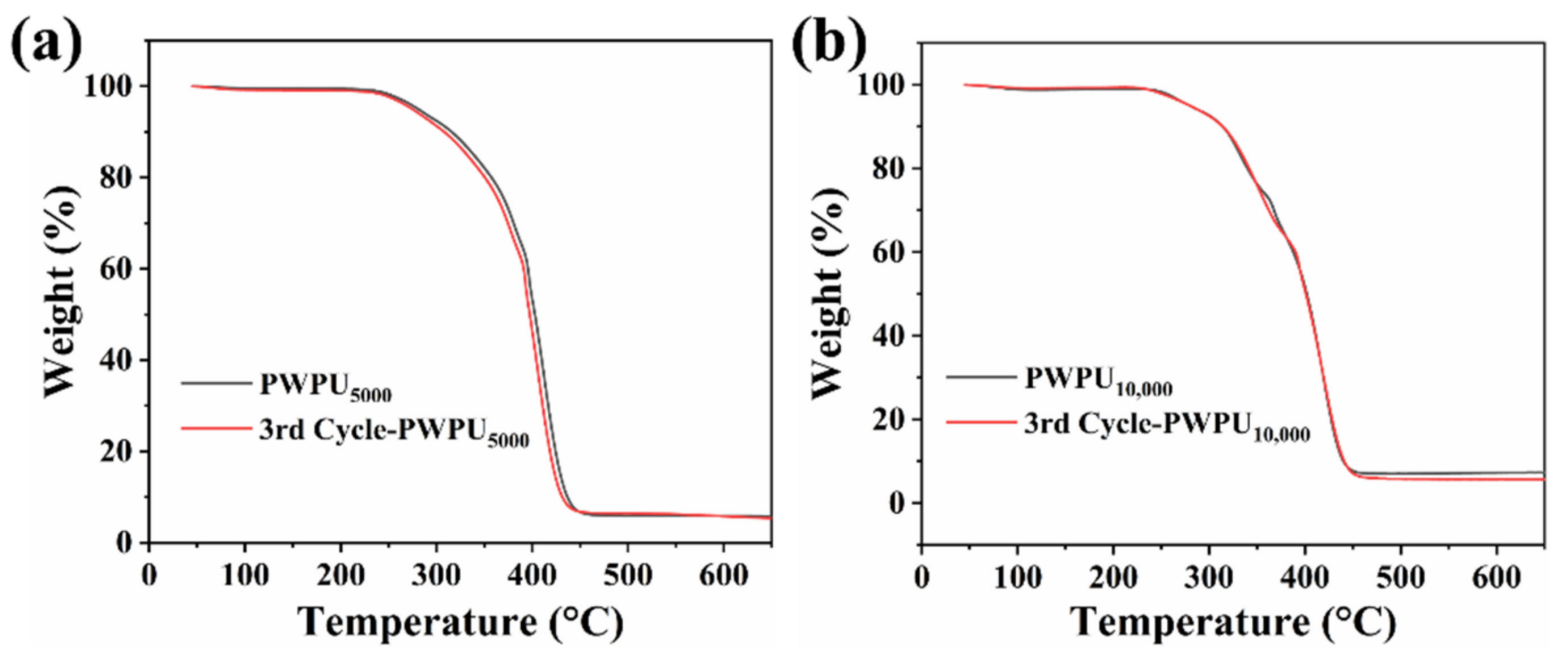
TGA curves of PWPU_5000_ (**a**) and PWPU_10,000_ (**b**) samples before and after three recycling processes.

Furthermore, the recycled PWPU film (recycled three times) with a thickness of 0.3 mm was capable of lifting even a weight of 10 kg (Figure 12d and Figure S10d), indicating superior mechanical properties of recycled PWPU. These results indicate that the PWPU film still maintained excellent mechanical properties after being recycled three times, which confirmed extraordinary recyclability of PWPU. The thermal weight loss curves of the recycled PWPU_5000_ and PWPU_10,000_ films are shown in Figure 13. Table 4 summarizes the results of the selected parameters obtained from the TGA thermograms. When comparing the thermal weight loss curves of the original PWPU film and the recycled PWPU film, it can be observed that PWPU_5000_ and PWPU_10,000_ still maintain good thermal stability after being recycled three times [39].

### 3.6. Shape-Memory Performance of PWPU_-X_

**Figure 14:**
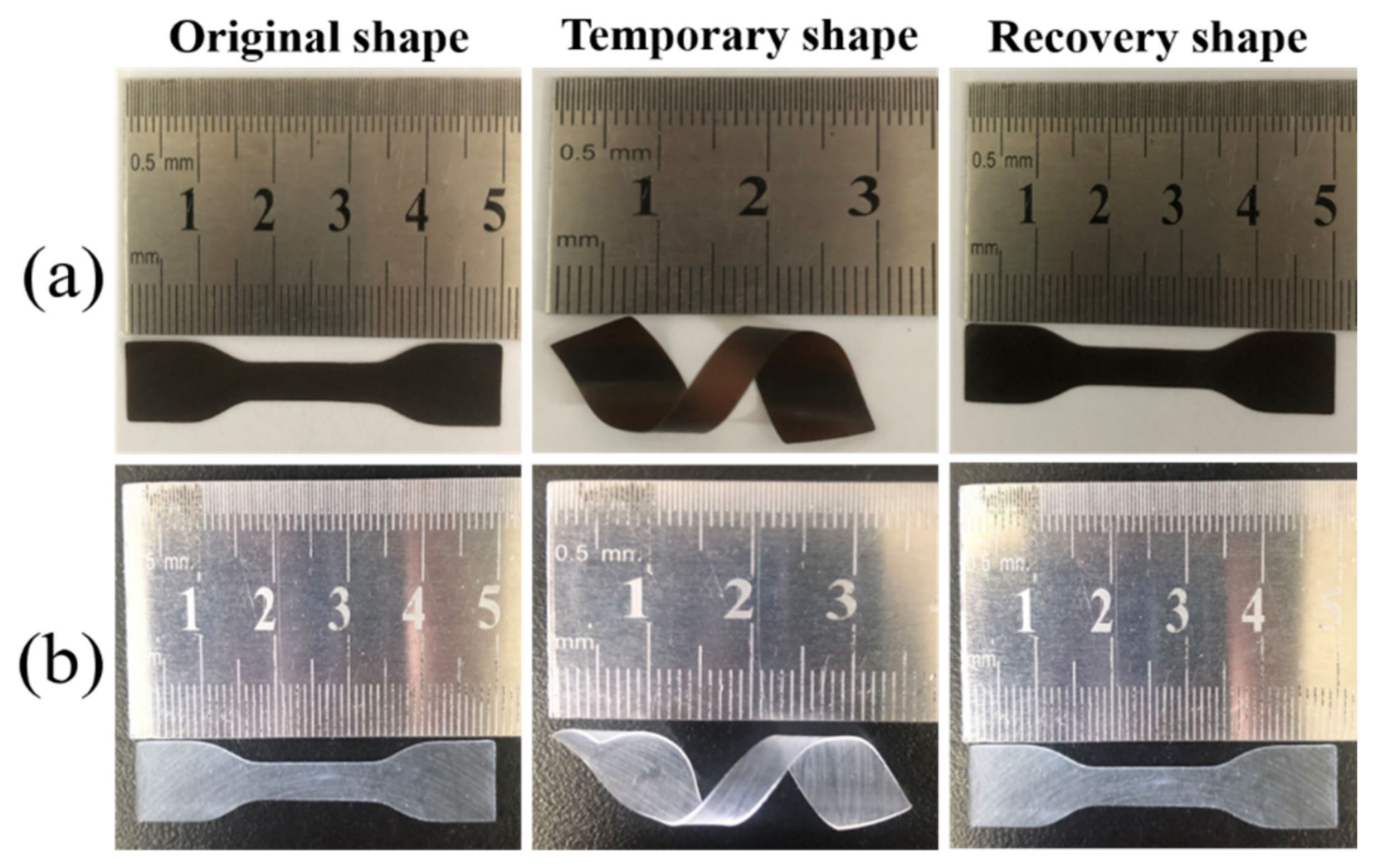
The shape-memory behavior of PWPU_5000_ (**a**) and BWPU_5000_ (**b**). Photos with original shape, temporary shape (deformed at 25 °C for 20 min and fixed at 0 °C for 20 min) and recovered state after being soaked in 70 °C water.

**Figure 15:**
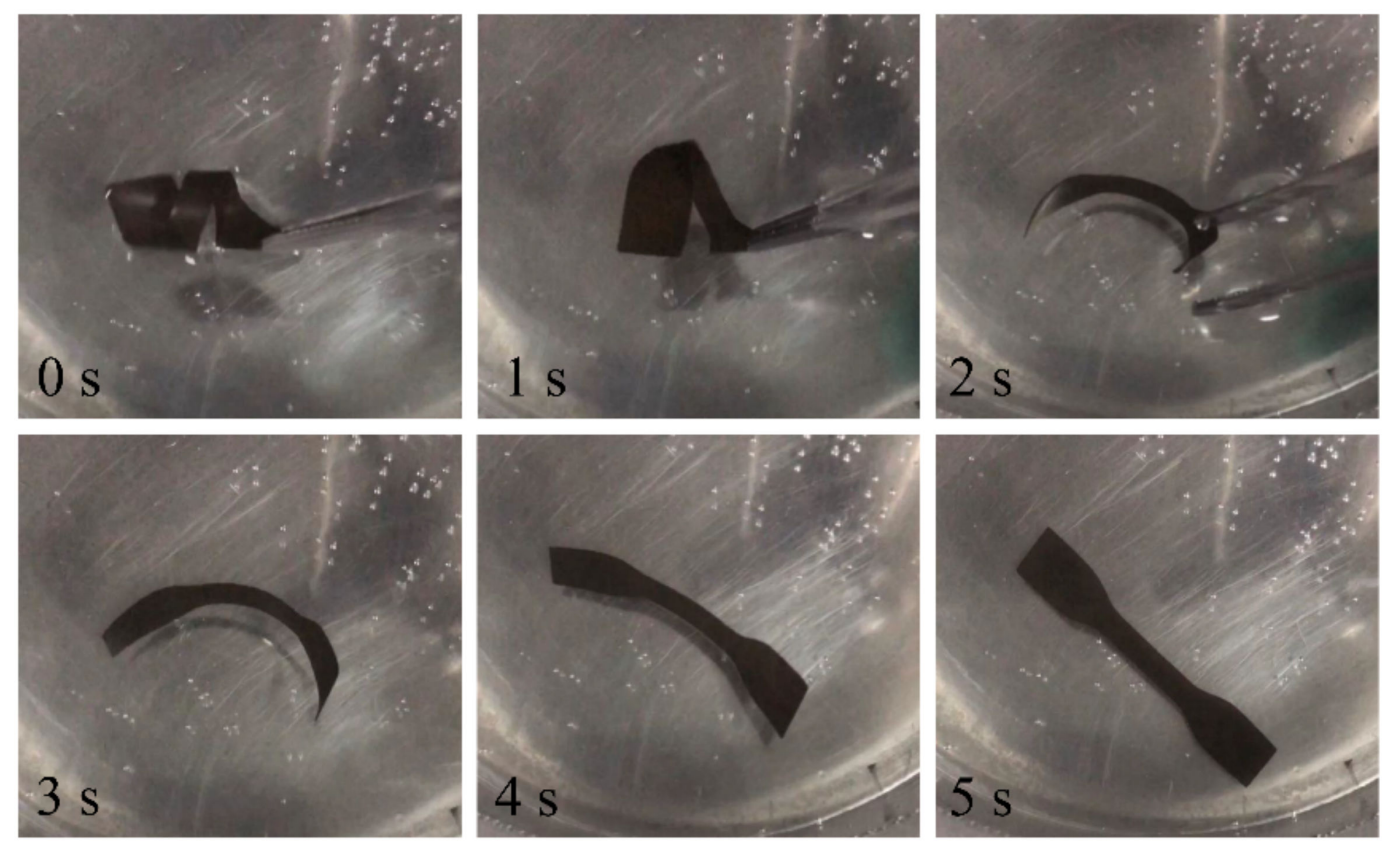
Photos of PWPU_5000_ shape recovery in water at 70 °C.

The soft and hard segments of waterborne polyurethane materials are thermodynamically incompatible and can easily form their separate microregions and undergo microphase separation [40]. It is the characteristic of the block structure of polyurethane materials that gives them their shape-memory function. During the shape-memory process, the physical crosslinking point provides the strength of the material. The soft segment acts as a shape-memory unit to fix the temporary shape [41]. AFM and DMA results indicate that BWPU and PWPU films exhibited a microphase separation structure. Therefore, they had potential shape-memory properties. First, the dumbbell-like film was made into a particular shape at 25 °C for 20 min under external force. Then, the film was placed in a 0 °C refrigerator for 20 min to get a temporary shape which recovered to its original state after being soaked in 70 °C water (Figure 14, Figure S11). Figure 15 shows that PWPU_5000_ recovered in 5 s in 70 °C water (Movie S1), and BWPU_5000_ took 8 s to recover in 70 °C water (Figure S12, Movie S2). After keeping the temporary shape for 20 min at 0 °C, heating the PWPU_10,000_ and BWPU_10,000_ to 70 °C, the temporary spiral shape recovered its original shape in 9 s (Movies S3 and S4). It can be observed that the introduction of PBI-PCL as a soft segment in waterborne polyurethane had a positive effect on the shape-memory function.

## 4. Conclusions

In this paper, a series of waterborne polyurethanes PWPU and BWPU were synthesized by tailoring the soft segment component, and their structures were characterized by ^1^H-NMR and FTIR. The DLS and TEM results indicate that the PWPU emulsions emerged with more uniform particle size distribution and good storage stability. TGA analysis and mechanical test results demonstrate that the mechanical properties and thermal stability of PWPU films were significantly higher than those of BWPU films. DMA and AFM tests revealed that PWPU films had remarkable microphase separation, making PWPU film swell and dissolve faster in acetone/ethanol (*v*/*v* = 2:1) at room temperature. More importantly, the dissolved PWPU could be dispersed in deionized water again to get a waterborne polyurethane emulsion. On the contrary, the BWPU films could not be recycled with this method. Furthermore, after being recycled three times, the thermal stability of PWPU emulsion and PWPU film remained unchanged. These results provide some insight into the fact that tailoring the soft segment structures in WPU results in optimizing different properties. The obtained waterborne polyurethanes have potential for application in recyclable coatings and high-performance polymers.

## Figures and Tables

**Table 1:** The formulations for PWPU_5000_, BWPU_5000_, PWPU_10,000_ and BWPU_10,000_.

Sample	IPDI (mmol)	PTMG_2000_ (mmol)	PBI-PCL_5000_ (mmol)	BDO-PCL_5000_ (mmol)	PBI-PCL_10,000_ (mmol)	BDO-PCL_10,000_ (mmol)	DMPA (mmol)	A95 (mmol)
PWPU_5000_	60	19.2	0.8	0	0	0	20	28
BWPU_5000_	60	19.2	0	0.8	0	0	20	28
PWPU_10,000_	60	19.2	0	0	0.8	0	20	28
BWPU_10,000_	60	19.2	0	0	0	0.8	20	28

**Table 2:** Physicochemical parameters of PWPU and BWPU emulsions (0.5 mg/mL).

Samples	Dz ^a^ (nm)	PDI ^b^	Zeta Potential ^c^ (mV)
BWPU_5000_	80.99	0.232	−54.5
PWPU_5000_	40.26	0.131	−60.7
BWPU_10,000_	153.3	0.254	−50.1
PWPU_10,000_	49.05	0.216	−54.0

^a^ Z-average diameter determined by DLS. ^b^ Polydispersity of particles determined by DLS. ^c^ Determined by DLS at 25 °C.

**Table 3:** Data of thermal decomposition of BWPU_5000_, PWPU_5000_, BWPU_10,000_ and PWPU_10,000_.

Samples	T_5%_/°C	T_10%_/°C	T_50%_/°C	T_80%_/°C
BWPU_5000_	261.2	296.1	390.3	418.3
PWPU_5000_	281.0	315.9	402.7	424.4
BWPU_10,000_	262.2	303.1	384.3	415.0
PWPU_10,000_	280.1	314.3	401.2	428.5

**Table 4:** Data of thermal decomposition.

Samples	T_5%_/°C	T_20%_/°C	T_50%_/°C	T_80%_/°C
PWPU_5000_	281.0	357.4	402.7	424.4
3rd Cycle-PWPU_5000_	274.8	350.6	397.4	418.5
PWPU_10,000_	280.1	341.6	401.2	428.5
3rd Cycle-PWPU_10,000_	279.8	341.6	400.9	428.3

## Data Availability

The data presented in this study are available on request from the corresponding author.

## Supplementary Materials

The following are available online at https://www.mdpi.com/article/10.3390/polym13111755/s1, Scheme S1: Synthesis of PBI-OH, Scheme S2: Synthesis of PBI-PCL_5000_ and PBI-PCL_10,000_, Scheme S3: Synthesis of BDO-PCL_5000_ and BDO-PCL_10,000_, Scheme S4: Synthesis of BWPU_5000_ and BWPU_10,000,_ Figure S1: ^1^H-NMR spectra of PBI, PBI-PCL_5000_ and PBI-PCL_10,000_, Figure S2: ^1^H-NMR spectra of BDO-PCL_5000_ and BDO-PCL_10,000_, Figure S3: FTIR spectra of BDO, BDO-PCL_10,000_, BWPU_10,000_ and PWPU_10,000,_ Figure S4: The zeta potential distribution of PWPU and BWPU emulsions, Figure S5: The dissolution images of PWPU_10,000_ under different solvents for 3 hours; (a) DMF; (b) NMP;(c) Acetone/Ethanol (*v/v* = 2:1) (d) Acetone; (e) Ethanol, Figure S6: The dissolution images of BWPU_10,000_ under different solvents for 3 hours; (a)DMF; (b)NMP;(c) Acetone/Ethanol (*v/v* = 2:1) (d) Acetone; (e) Ethanol, Figure S7: Photos of BWPU_5000_ (a) and PWPU_5000_ (b) with a mass of 0.5 g dissolved in 4 mL of acetone/ethanol mixed-solvent (*v/v* = 2:1) at 25 °C for 3 h, Figure S8: Photos of BWPU_10,000_ (a) and PWPU_10,000_ (b) with a mass of 0.5 g dissolved in 4 mL of acetone/ethanol mixed-solvent (*v/v* = 2:1) at 25 °C for 3 h, Figure S9: Zeta potential distribution of recycled PWPU_5000_ (a) and PWPU_10,000_ (b) emulsions, Figure S10: (a) Stress**–**strain curves of PWPU_5000_ after three-generations of recycling. The mechanical properties including the (b) breaking strength, and (c) breaking elongation for the PWPU_5000_ samples after three times recycling process. (d) Photo of lifting a 10 kg weight by a PWPU_5000_ film with the thickness of 0.3 mm after the recycling process, Figure S11: The shape memory behavior of BWPU_10,000_ (a) and PWPU_10,000_ (b) photos with original shape, temporary shape (deformed at 25 °C for 20 min and fixed at 0 °C for 20 min), and recovered state after being soaked in water at 70 °C, Figure S12: Photos of BWPU_5000_ shape recovery in water at 70 °C, Movie S1: Video of PWPU_5000_ shape recovery in water at 70 °C, Movie S2: Video of BWPU_5000_ shape recovery in water at 70 °C, Movie S3: Video of PWPU_10,000_ shape recovery in water at 70 °C, Movie S4: Video of BWPU_10,000_ shape recovery in water at 70 °C.
