# Supplementary material for: Recyclable Shape-Memory Waterborne Polyurethane Films Based on Perylene Bisimide Modified Polycaprolactone Diol
Source: Polymers (Basel). 2021 May 27;13(11):1755. doi: 10.3390/polym13111755 (PMC8198087; doi:10.3390/polym13111755)
Supplement: Supplementary file 1 [file polymers-13-01755-s001.zip › polymers-1212435-supplementary.pdf]

Supporting Information

Article

Recyclable shape-memory waterborne polyurethane films based on perylene bisimide modified polycaprolactone diol

Kang Wei [1], Haitao Zhang [1,2], Jianbo Qu [1], Jianyong Wang [1], Yang Bai [3] and Futao Sai [1]

[1] School of Light Industry Science and Engineering, Qilu University of Technology, Shandong Academy of Sciences, Jinan, 250353, China; wk180203@163.com (K.W.); qujianbo396@sohu.com (J.Q.); wjy@qlu.edu.cn (J.W.); 17864181422@163.com (F.S.)

[2] State Key Laboratory of Biobased Material and Green Papermaking, Qilu University of Technology, Shandong Academy of Sciences, Jinan, 250353, China.

[3] Shaanxi Key Laboratory of Chemical Additives for Industry, College of Chemistry and Chemical Engineering, Shaanxi University of Science and Technology, Xi'an 710021.

Correspondence: zhanghaitao@qlu.edu.cn (H.Z.), Tel:+86-133-6531-6033; baiyang@sust.edu.cn (Y.B.)Tel: +86-19-9186-0664.

Table of Contents

1. **Experimental section**

   1.1 Synthesis of PBI-OH

   1.2 Synthesis of $PBI\text{-}PCL_{5000}$ and $PBI\text{-}PCL_{10,000}$

   1.3 Synthesis of $BDO\text{-}PCL_{5000}$ and $BDO\text{-}PCL_{10,000}$

   1.4 Synthesis of $PWPU_{5000}$, $PWPU_{10,000}$, $BWPU_{5000}$ and $BWPU_{10,000}$

2. **Additional experimental results**

   2.1 ^{1}H-NMR spectra of PBI, $PBI\text{-}PCL_{5000}$ and $PBI\text{-}PCL_{10,000}$

   2.2 ^{1}H-NMR spectra of $BDO\text{-}PCL_{5000}$ and $BDO\text{-}PCL_{10,000}$

   2.3 FTIR spectra of BDO, $BDO\text{-}PCL_{10,000}$, $BWPU_{10,000}$ and $PWPU_{10,000}$

   2.4 The zeta potential distribution of PWPU and BWPU emulsions

3. **Additional experimental results of recyclable performance analysis**

   3.1 Screen the recycled solvent

   3.2 The zeta potential distribution of recycled $PWPU_{5000}$ and $PWPU_{10,000}$

emulsions

3.3 Stress–strain curves of recycled $PWPU_{5000}$ film

4. Additional experimental results of shape memory

1. Experimental Section

1.1 Synthesis of PBI-OH

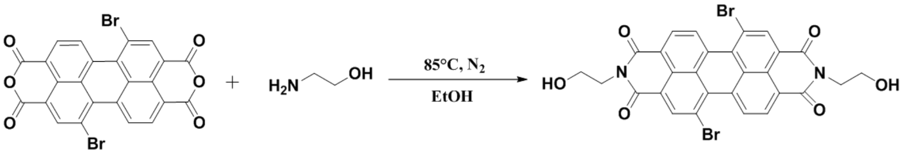

Scheme S1 Synthesis of PBI-OH.

1,7-dibromo-3,4,9,10-per tetracarboxylic dianhydride (1 g, 1.82 mmol), ethanolamine (0.334 g, 5.46 mmol) and absolute ethanol (15 ml) were placed in a three-necked flask equipped with a magnet stirrer under nitrogen atmosphere at 85°C for 5 h. After the reaction, the mixture was cooled to room temperature and diluted with ethanol (50 ml). The precipitate was obtained through centrifugation and purified with cooled ethanol (4×20 ml). Finally, the products were dried for 24 h at 60°C in vacuum and 0.9807 g of product was obtained with yield of 86%. ^{1}H-NMR (400 MHz, CDCl3): δ=9.59 (2H, d, phenyl–H), 8.98 (2H, s, phenyl–H), 8.78 (2H, d, phenyl–H), 4.60 (4H, s, –CH_2–), 4.22(4H, s, –CH_2–).

1.2 Synthesis of PBI-PCL$_{5000}$ and PBI-PCL$_{10,000}$

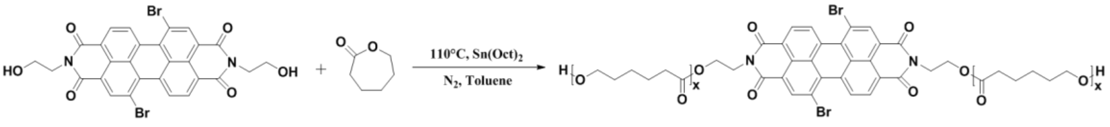

Scheme S2 Synthesis of PBI-PCL$_{5000}$ and PBI-PCL$_{10,000}$.

The PBI-PCL (PBI-PCL$_{5000}$ and PBI-PCL$_{10,000}$, where the number indicates the molecular weight of poly(ε- caprolactone) (PCL)) were synthesized from PBI-OH and ε-CL through controlling the content of ε-CL. PBI-PCL$_{5000}$ was chosen as an example. In a typical reaction, PBI-OH (2 g, 3.14 mmol), ε-CL (14.35 g, 125.74 mmol), $Sn(Oct)_2$ (0.1435g, as catalyst) and anhydrous toluene (30 ml) were mixed into a three-necked round bottom flask. The mixture was vigorously stirred at room temperature for 5 min and then stirred at 110°C for another 20 h under dry nitrogen. After that, After the reaction, the mixture was cooled to room temperature and diluted with ethanol (150 ml). The precipitate was obtained through centrifugation and purified with cooled methanol (4×30 ml). The product was obtained after drying in vacuum oven at 40°C for 24 h and 15.12 g of product was obtained with yield of 89%. ^{1}H-NMR (400 MHz, $CDCl_3$) δ=9.51 (2H, d, phenyl–H), 8.93 (2H, d, phenyl–H), 8.71 (2H, s, phenyl–H), 4.48 (4H, s, –CH_2–), 4.24 (4H, s, –CH_2–), 4.06 (84H, t, –CH_2), 3.65 (4H, t, –CH_2–OH), 2.31 (84H, t, –CO–CH_2–), 1.65 (168H, m, –CH_2), 1.38 (84H, t, –CH_2). PBI-PCL$_{10,000}$ was prepared in the same manner.

1.3 Synthesis of BDO-PCL$_{5000}$ and BDO-PCL$_{10,000}$

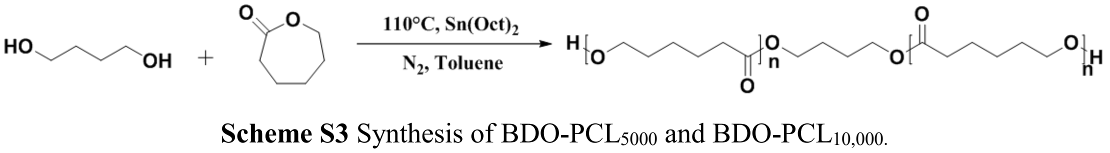

Scheme S3 Synthesis of BDO-PCL$_{5000}$ and BDO-PCL$_{10,000}$.

BDO-PCL$_{5000}$ and BDO-PCL$_{10,000}$ was prepared in the same manner as PBI-PCL$_{5000}$ and PBI-PCL$_{10,000}$.

1.4 Synthesis of PWPU$_{5000}$, PWPU$_{10000}$, BWPU$_{5000}$ and BWPU$_{10,000}$

In a typical procedure of BWPUs and PWPUs (BWPU$_{5000}$, BWPU$_{10,000}$, PWPU$_{5000}$ and PWPU$_{10,000}$ where the number indicates the corresponding PCL used), PWPU$_{5000}$ was chosen as an

example. In a typical reaction, PBI-PCL_{5000} (4 g, 0.8 mmol), $PTMG_{2000}$ (38.4 g, 19.2 mmol) and IPDI (13.32 g, 60 mmol) were added into a dry three-neck flask equipped with a mechanical stirrer, 0.05 ml of organobism bismuth catalyst was added and stirred in an oil bath at 86°C for 1.5 h. After DMPA (2.76 g, 20 mmol) was added and the reaction was continued for 2.5 h, an appropriate amount of acetone was added to the system to reduce its viscosity. When the reaction mixture was slowly cooled to 45°C, TEA (2.08 g, 20 mmol) was added to the system to neutralize the side chain carboxyl groups, and finally the polymer was added to 120 ml deionized water and the solution was emulsified by stirring at high shear speed, and finally A95 was added dropwise to post-expand the chain to obtain PWPU emulsion. $PWPU_{10,000}$, $BWPU_{5000}$ and $BWPU_{10,000}$ were obtained using PBI-$PCL_{10,000}$, BDO-PCL_{5000} and BDO-$PCL_{10,000}$ in the same way.

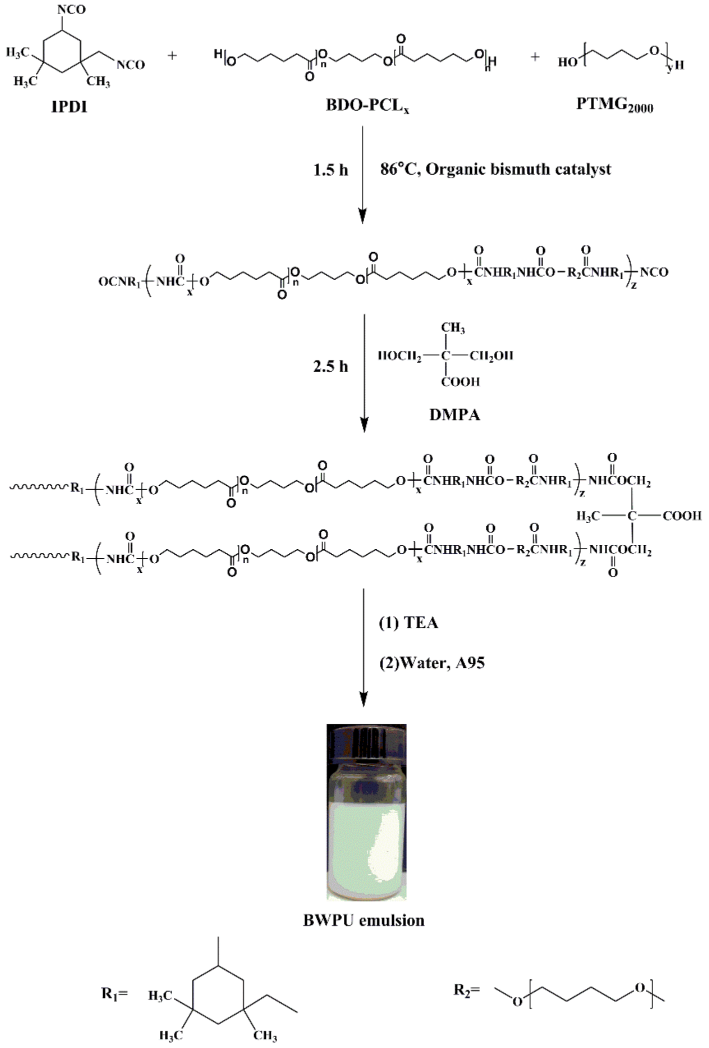

Scheme S4 Synthesis of $BWPU_{5000}$ and $BWPU_{10,000}$.

2. Additional Experimental Results

2.1 ^{1}H-NMR Spectra of PBI, PBI-PCL$_{5000}$ and PBI-PCL$_{10,000}$

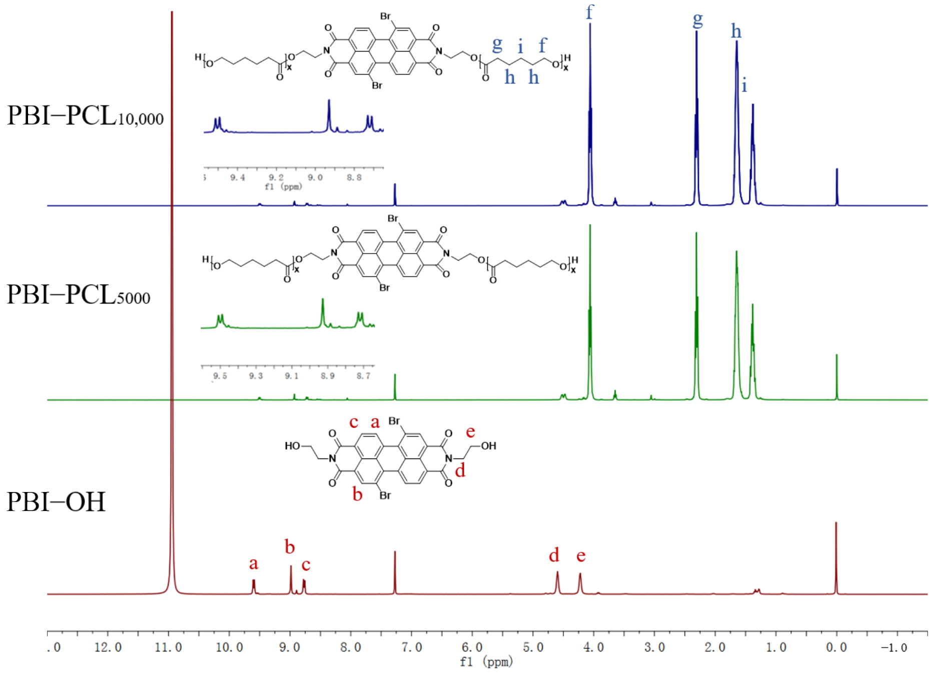

Figure S1 ^{1}H-NMR spectra of PBI, PBI-PCL$_{5000}$ and PBI-PCL$_{10,000}$.

2.2 ^{1}H-NMR Spectra of BDO-PCL$_{5000}$ and BDO-PCL$_{10,000}$

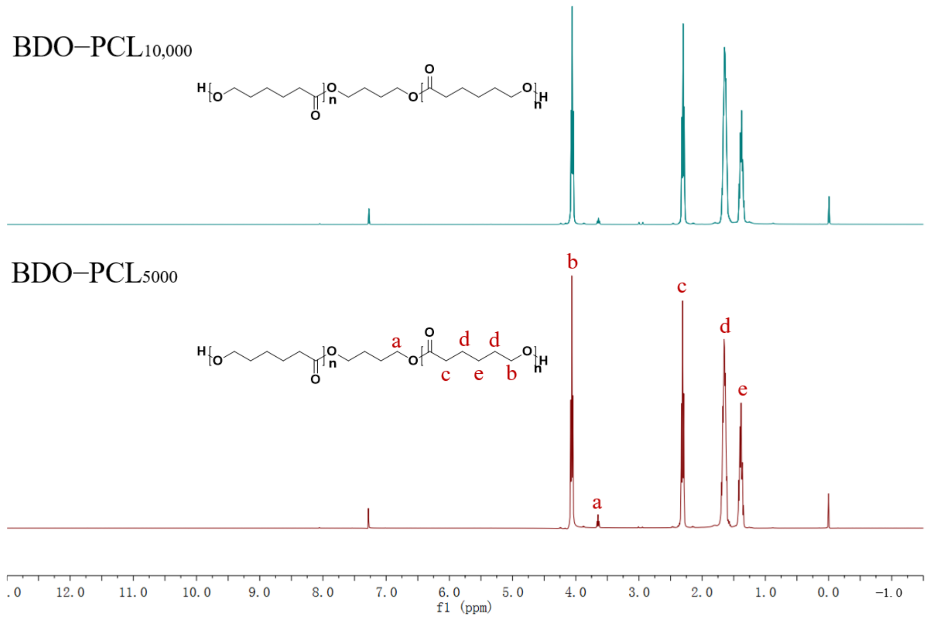

Figure S2 ^{1}H-NMR spectra of BDO-PCL$_{5000}$ and BDO-PCL$_{10,000}$.

2.3 FTIR spectra of BDO, BDO-PCL$_{10,000}$, BWPU$_{10,000}$ and PWPU$_{10,000}$

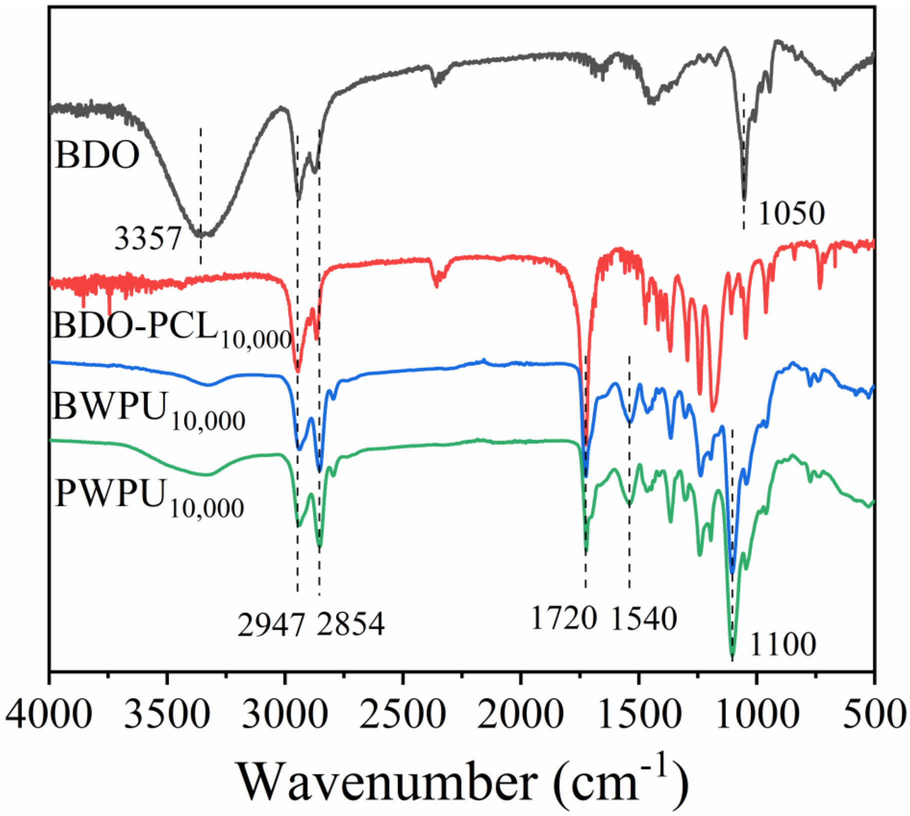

Figure S3. FTIR spectra of BDO, BDO-PCL$_{10,000}$, BWPU$_{10,000}$ and PWPU$_{10,000}$.

2.4 The Zeta Potential Distribution of PWPU and BWPU Emulsions

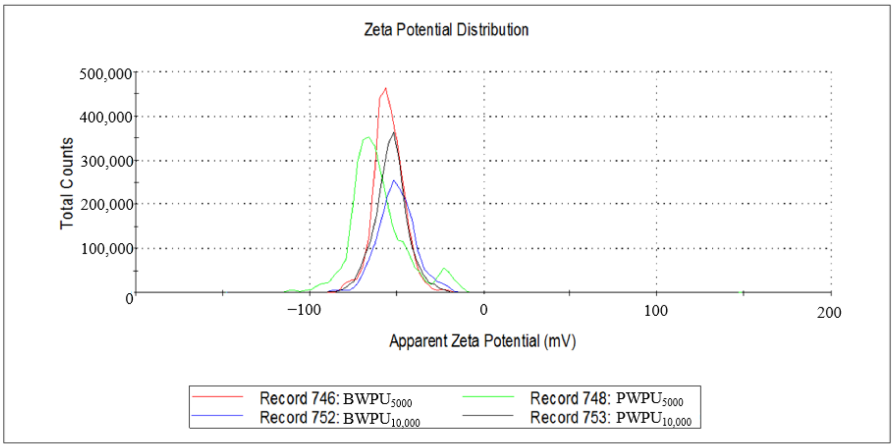

Figure S4 The zeta potential distribution of PWPU and BWPU emulsions.

3. Additional Experimental Results of Recyclable Performance Analysis

3.1 Screen the Recycled Solvent

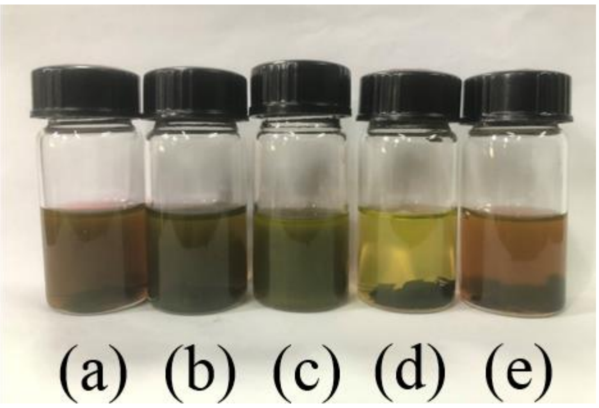

Figure S5 The dissolution images of $PWPU_{10,000}$ under different solvents for 3 hours; (a)DMF; (b)NMP;(c) Acetone/Ethanol (*v/v* = 2:1) (d) Acetone; (e) Ethanol.

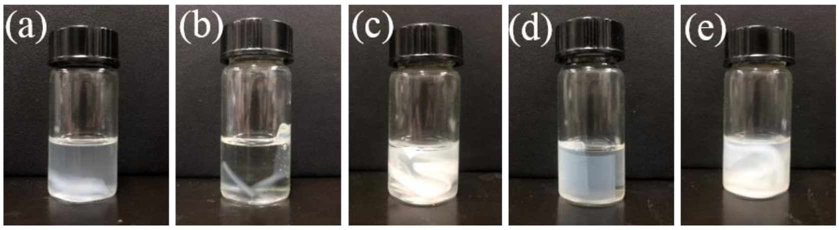

Figure S6 The dissolution images of $BWPU_{10,000}$ under different solvents for 3 hours; (a)DMF; (b)NMP;(c) Acetone/Ethanol (*v/v*=2:1) (d) Acetone; (e) Ethanol.

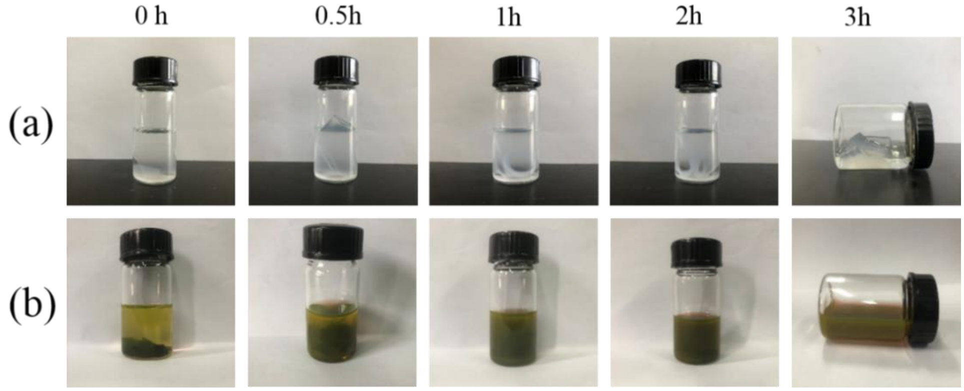

Figure S7 Photos of $BWPU_{5000}$ (a) and $PWPU_{5000}$ (b) with a mass of 0.5 g dissolved in 4 ml of acetone/ethanol mixed-solvent (*v/v*=2:1) at 25°C for 3 h.

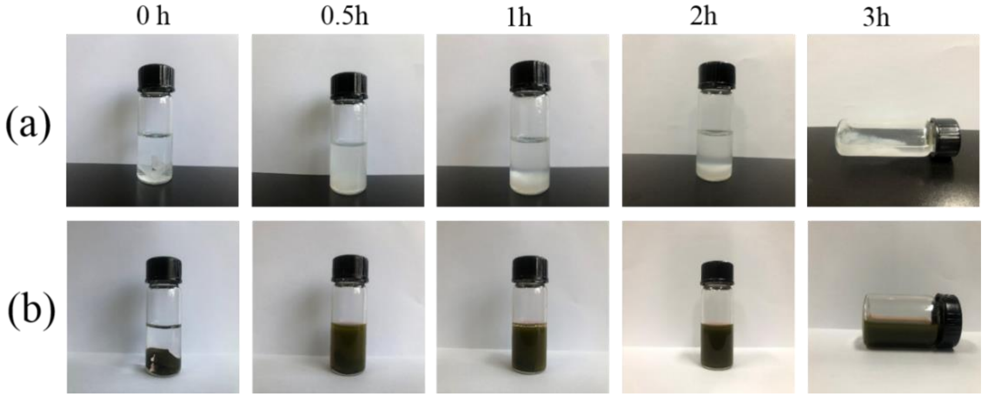

Figure S8 Photos of $BWPU_{10,000}$ (a) and $PWPU_{10,000}$ (b) with a mass of 0.5 g dissolved in 4 ml of acetone/ethanol mixed-solvent (*v/v*=2:1) at 25°C for 3 h.

3.2 The Zeta Potential Distribution of Recycled $PWPU_{5000}$ and $PWPU_{10,000}$ Emulsions

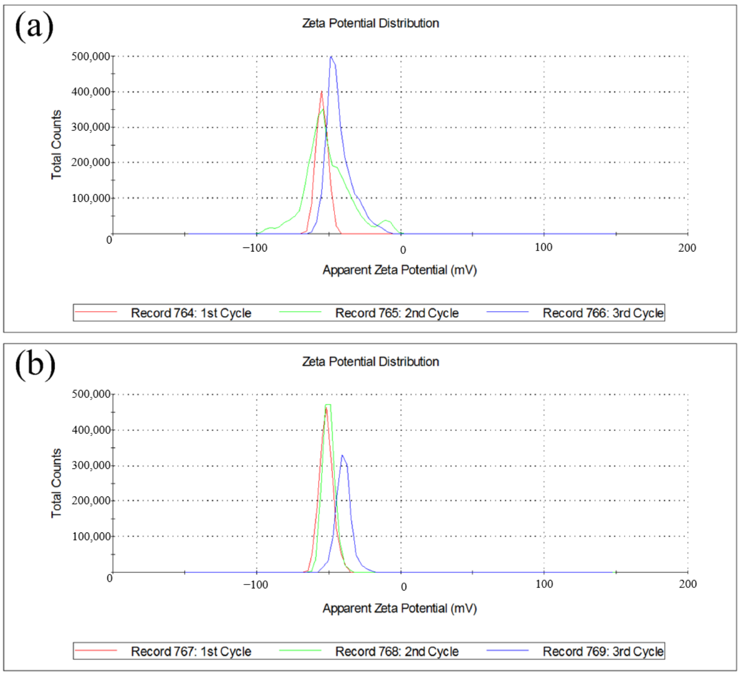

Figure S9 Zeta potential distribution of recycled $PWPU_{5000}$ (a) and $PWPU_{10,000}$ (b) emulsions.

3.3 Stress–strain Curves of Recycled $PWPU_{5000}$ Film

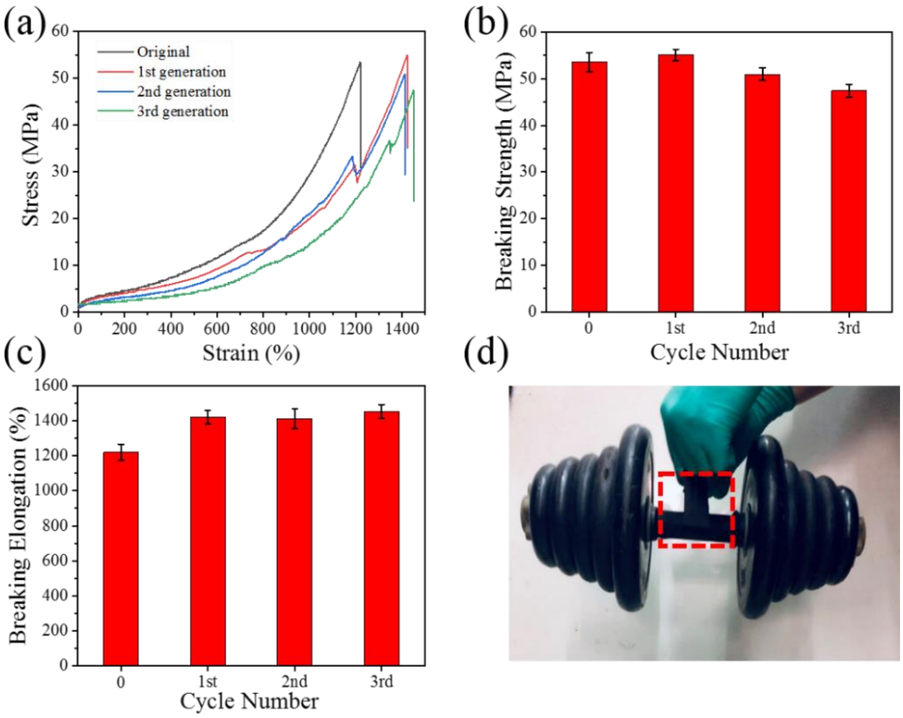

Figure S10 (a) Stress–strain curves of $PWPU_{5000}$ after three-generations of recycling. The mechanical properties including the (b) breaking strength, and (c) breaking elongation for the $PWPU_{5000}$ samples after three times recycling process. (d) Photo of lifting a 10 kg weight by a $PWPU_{5000}$ film with the thickness of 0.3 mm after the recycling process.

4. Additional Experimental Results of Shape Memory

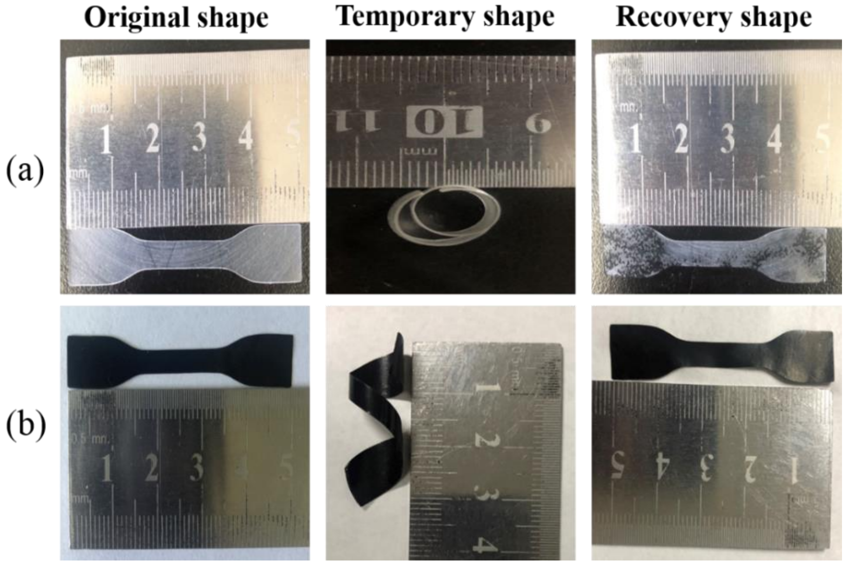

Figure S11 The shape memory behavior of $BWPU_{10,000}$ (a) and $PWPU_{10,000}$ (b) photos with original shape, temporary shape (deformed at 25°C for 20 min and fixed at 0°C for 20 min), and recovered state after being soaked in water at 70°C.

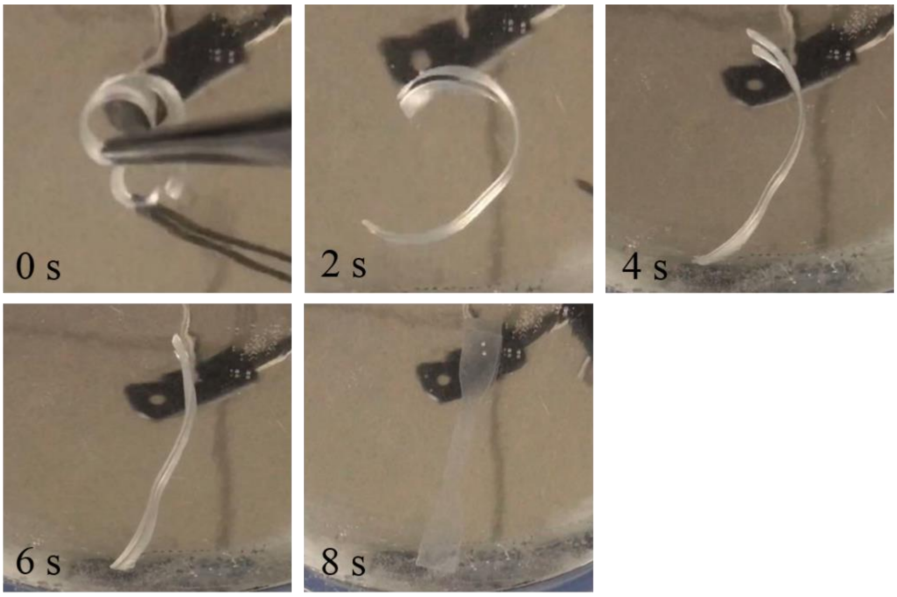

Figure S12 Photos of $BWPU_{5000}$ shape recovery in water at 70°C.
